# The causal effect of cytokine cycling levels on osteoarthritis: a bidirectional Mendelian randomized study

**DOI:** 10.3389/fimmu.2023.1334361

**Published:** 2024-01-11

**Authors:** Zong Jiang, Xin Cai, Xiaoling Yao, Shaoqin Zhang, Weiya Lan, Zexu Jin, Fang Tang, Wukai Ma, Xueming Yao, Changming Chen, Tianzuo Lan

**Affiliations:** ^1^ Second Clinical Medical College, Guizhou University of Traditional Chinese Medicine, Guiyang, China; ^2^ Department of Rheumatology and Immunology, The First People's Hospital Of Guiyang, Guiyang, China; ^3^ Department of Rheumatology and Immunology, The Second Affiliated Hospital of Guizhou University of Traditional Chinese Medicine, Guiyang, China

**Keywords:** cytokines, osteoarthritis, Mendelian randomization, bidirectional, meta analysis

## Abstract

**Objective:**

Osteoarthritis (OA) is the most prevalent joint disease globally, serving as a primary cause of pain and disability. However, the pathological processes underlying OA remain incompletely understood. Several studies have noted an association between cytokines and OA, yet the causal link between them remains ambiguous. This study aims to identify cytokines potentially causally related to OA using Mendelian randomization (MR) analysis, informing early clinical diagnosis and treatment decisions.

**Methods:**

We conducted a genome-wide association study (GWAS) on 12 OA traits involving 177,517 cases and 649,173 controls from 9 international cohorts. For discovery MR analysis, we used 103 cytokines from two European populations as instrumental variables (IVs). Concurrently, another European population OA GWAS database (36,185 cases and 135,185 controls) was used to replicate MR analysis, employing the inverse variance weighted (IVW) method as the primary analytic approach. Additional methods tested included MR Egger, Weighted median, and Weighted mode. We merged the MR findings through meta-analysis. Heterogeneity testing, level pleiotropy testing (MR Egger intercept test and MRPRESSO), and sensitivity analysis via Leave One Out (LOO) were conducted to verify result robustness. Lastly, reverse MR analysis was performed.

**Results:**

The meta-analysis merger revealed a correlation between CX3CL1 cycle levels and increased OA risk (OR=1.070, 95% CI: 1.040-1.110; P<0.010). We also observed associations between MCP4 (OR=0.930, 95% CI: 0.890-0.970; P<0.010) and CCL25 (OR=0.930, 95% CI: 0.890-0.970; P<0.010) with reduced OA risk. The sensitivity analysis results corroborate the robustness of these findings.

**Conclusion:**

Our MR analysis indicates a potential causal relationship between CX3CL1, MCP4, CCL25, and OA risk changes. Further research is warranted to explore the influence of cytokines on OA development.

## 1 Introduction

Osteoarthritis (OA) is a chronic degenerative condition marked by osteochondral degradation, manifesting as joint pain, stiffness, swelling, and reduced joint mobility. It predominantly impacts weight-bearing joints like the knee, hip, and spine (1, 2). Statistically, OA affects over 520 million individuals globally (3). with its prevalence escalating alongside population aging, significantly impairing patient quality of life (4). The etiology of OA remains elusive, with studies suggesting its association with factors like genetic variation, mechanical damage, immune inflammatory response, and metabolic irregularities (5, 6). Given the uncertain etiology, there is no definitive cure for OA. Currently, treatments involve general physical therapy to slow disease progression, non-steroidal anti-inflammatory drugs for symptom relief, and agents to promote joint cartilage repair. However, these long-term treatment strategies often yield suboptimal results. Post-joint replacement surgery, many patients continue to experience joint symptoms and are at risk of complications like cardiovascular events and infections (7). Thus, understanding OA’s pathogenesis and its early prevention are crucial. Presently, research primarily focuses on cartilage degeneration as a central aspect of OA’s pathogenesis, yet the underlying causes remain obscure. Clinical and basic research on cartilage remains pivotal in OA prevention and treatment (8, 9). However, the acquisition of research specimens and the associated costs are considerable challenges. Therefore, identifying biochemical biomarkers for OA prevention and treatment could represent a significant advancement.

Cytokines are small molecule proteins secreted and synthesized by immune cells like macrophages, monocytes, and T cells, as well as by non-immune cells such as fibroblasts and epidermal cells. They play a critical role in regulating immunity, stimulating cell activation and proliferation, and promoting hematopoiesis, thus being vital to human cellular function (10). Cytokines also contribute to diseases like inflammation, autoimmune disorders, and tumors (11, 12).

Increasing evidence indicates that the inflammatory response, mediated by inflammatory cytokines produced by synovial cells and chondrocytes in OA, is a key factor in OA pathogenesis. This response leads to cartilage matrix destruction and chondrogenesis inhibition (13). Immune cells activation, such as macrophages and lymphocytes, results in the production of tumor necrosis factor-α (TNF-α), interleukin-1β (IL-1β), and other inflammatory cytokines like interleukin-6 (IL-6). These cytokines exacerbate synovial inflammation, stimulate matrix metalloproteinases and oxidative stress, leading to chondrocyte apoptosis and cartilage degradation (14). Additionally, osteolytic cytokines like osteoclast activating factor (RANKL) play a role in OA development (15). However, the precise relationship between OA and the elevation of various cytokines remains unclear. Determining cytokine level alterations can further clarify their association with OA, providing a foundation for disease diagnosis and treatment.

Mendelian Randomization (MR) utilizes genome-wide association studies (GWASs), comprising single nucleotide polymorphisms (SNPs) from the population as genetic instrumental variables (IVs) to assess the causal links between exposure factors and outcomes (16, 17). These genetic variations, randomly assigned at conception, allow MR to circumvent confounding and reverse causal effects. Adhering to the genetic principle of “single gene single phenotype,” MR yields more reliable results and is extensively applied in analyzing relationships between various exposures and diseases (18). Current GWASs data includes IVs for over 100 cytokines. To date, only 2 MR studies have focused on OA, with a limited range of cytokines examined (18, 19). The incidence rate of OA varies across different body parts, and previous MR studies have not only examined a narrow scope of cytokines but also lacked detailed OA classifications. Thus, comprehensive research is necessary to elucidate the causal relationship between circulating cytokine levels and more specific OA categories, advancing our understanding of OA pathogenesis.

Therefore, this study conducts a bidirectional MR analysis based on publicly available GWAS data, employing the most comprehensive set of cytokine and OA data available. This approach aims to provide new insights into the causal relationship between cytokines and OA.

## 2 Materials and methods

### 2.1 Study design

**Figure 1:**
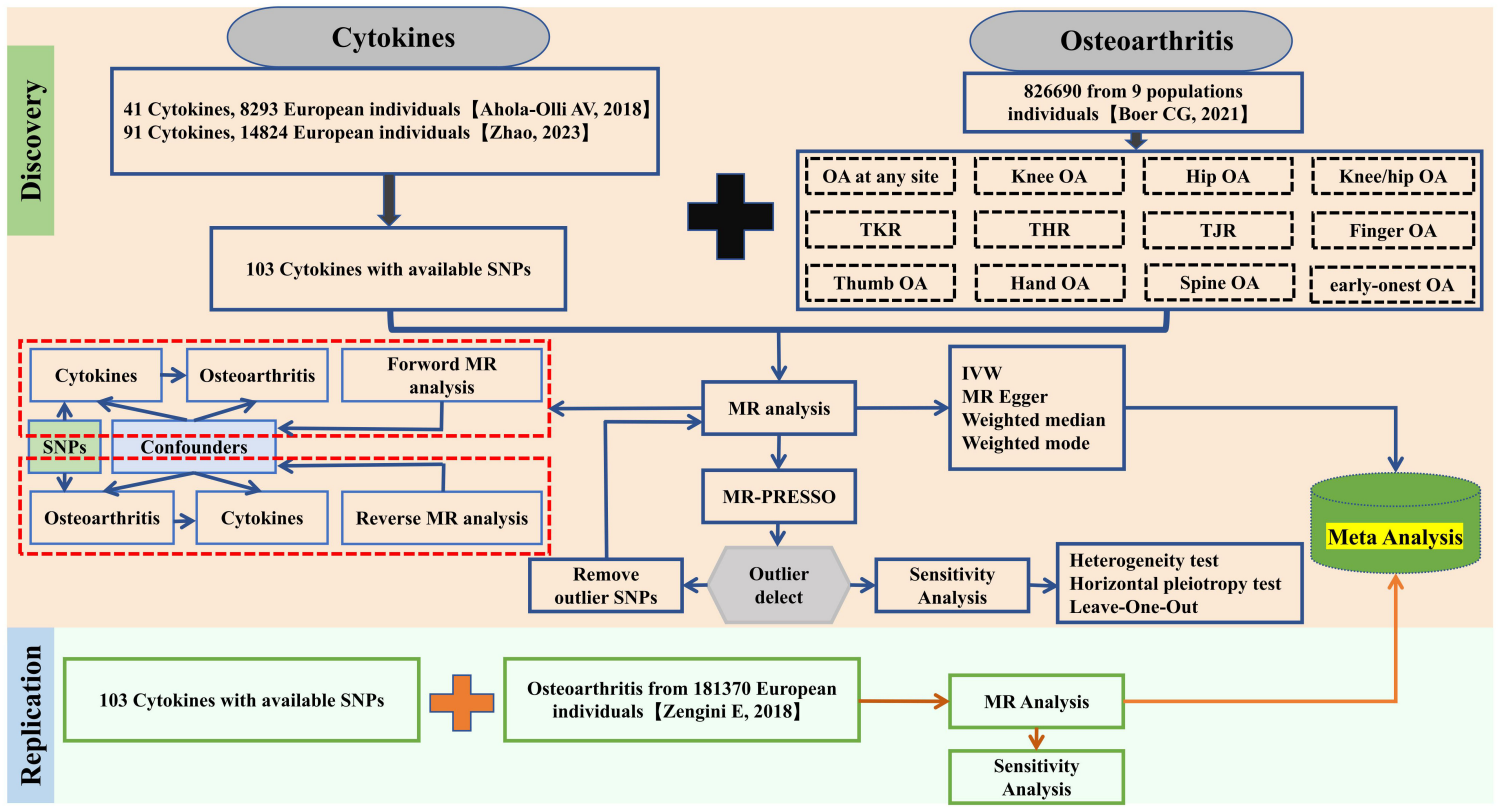
Study design and workflow of this study.

This research executed a broad and in-depth bidirectional MR analysis to explore a possible causal connection between cytokines and OA. The schematic of our MR analysis and design is depicted in **Figure 1**. MR studies must satisfy three criteria: first, the SNPs of the selected IVs must be significantly associated with cytokines. Second, these IVs should be independent and free from other confounding factors. Third, the IVs must influence outcomes exclusively through the exposure factors and not via alternate biological pathways. As this study utilizes publicly available GWAS data, no additional ethical approval was required.

### 2.2 Source of cytokine data

Cytokine data were sourced from two GWAS datasets (20, 21). Currently, 41 cytokines are commonly utilized in research investigating the correlation between cytokines and diseases. However, the latest GWAS data related to inflammation-related cytokines provide up to 91 kinds of cytokines. This extensive dataset offers more opportunities for investigating the association between inflammatory factors and osteoarthritis. To acquire a broader range of cytokines, this study initially selected GWAS data of 91 inflammation-related cytokines from 11 cohorts comprising 14,824 European individuals and supplemented this with GWAS data from 13 out of 41 cytokines from 8,293 European individuals, as these cytokines did not overlap with the aforementioned 91 cytokines. Additional detailed information about the aggregated GWAS data can be found in the original paper. In the principal analysis, cytokines with independent SNPs were isolated using a P-value threshold of <5×10^-8. Due to the limited number of cytokines reaching genome-wide significance, the P-value threshold was subsequently adjusted to <5×10^-6. Linkage disequilibrium analysis (LDA) was carried out to assure SNP independence, with criteria set at r^2<0.001 and kb=10,000.

### 2.3 Source of MR analysis data for osteoarthritis discovery

Data for OA were extracted from the most comprehensive GWAS database currently available, featuring 12 OA-related traits pooled from 9 international cohorts, totaling 826,690 participants (177,517 OA patients) across Asia, Europe, and European Americans (**Supplementary Table 1**) (22). Further information is detailed in the original publications. MR analysis was conducted on these OA-related traits, and the P-value threshold for OA-related SNPs was set at <5×10^-6, with concurrent LDA to ensure SNP independence (r^2<0.001 and kb=10,000).

### 2.4 Source of MR analysis data for the replication of osteoarthritis

To bolster the validity of the study outcomes and mitigate potential false positives, a supplementary dataset of 181,370 individuals (36,185 OA patients) was procured from the GWAS database, encompassing five OA traits for replication analyses (23). Similar to the primary study, a P-value threshold of <5×10^-6 was used to isolate cytokine SNPs, and LDA was performed to ascertain SNP independence, with criteria set at r^2<0.001 and kb=10,000.

### 2.5 Statistical analysis

Upon extracting SNP data for cytokines, we initially calculated the F-statistic for cytokines to assess the strength of the IVs (F>10 denotes sufficient strength) (24). All bidirectional MR analyses were conducted using the “TwoSampleMR” software package, with inverse variance weighting (IVW) as the primary, more precise, and unbiased method. Additionally, MR Egger, weighted median, and weighted mode analyses were employed to rule out potential confounders (25). For MR analysis outcomes, the false discovery rate (FDR) corrected p-values were computed using the Benjamin Hochberg method. A p-value below 0.05 post-FDR adjustment indicated a significant correlation. The findings from Discovery and replication MR analyses were then combined for a meta-analysis, integrating at least two reliably identified cytokine types. A merged P-value under 0.05 was deemed significant, with interpretations based on odds ratios (ORs).

### 2.6 Sensitivity analysis

To determine result robustness, we conducted heterogeneity testing, level pleiotropy testing, and Leave One Out (LOO) sensitivity analysis. Initially, MR-PRESSO was used to detect horizontal pleiotropy. If detected, outliers were removed for a subsequent MR reanalysis. If absent, the Cochran Q test was applied, with a Q value over 0.05 indicating no heterogeneity. LOO analysis was used to evaluate each SNP’s impact and identify any outliers. All MR analyses were performed using the “TwoSampleMR” and “MRPRESSO” software packages in R software (version 4.2.0) (26, 27).

## 3 Results

### 3.1 Discovery on the risk of osteoarthritis

**Figure 2:**
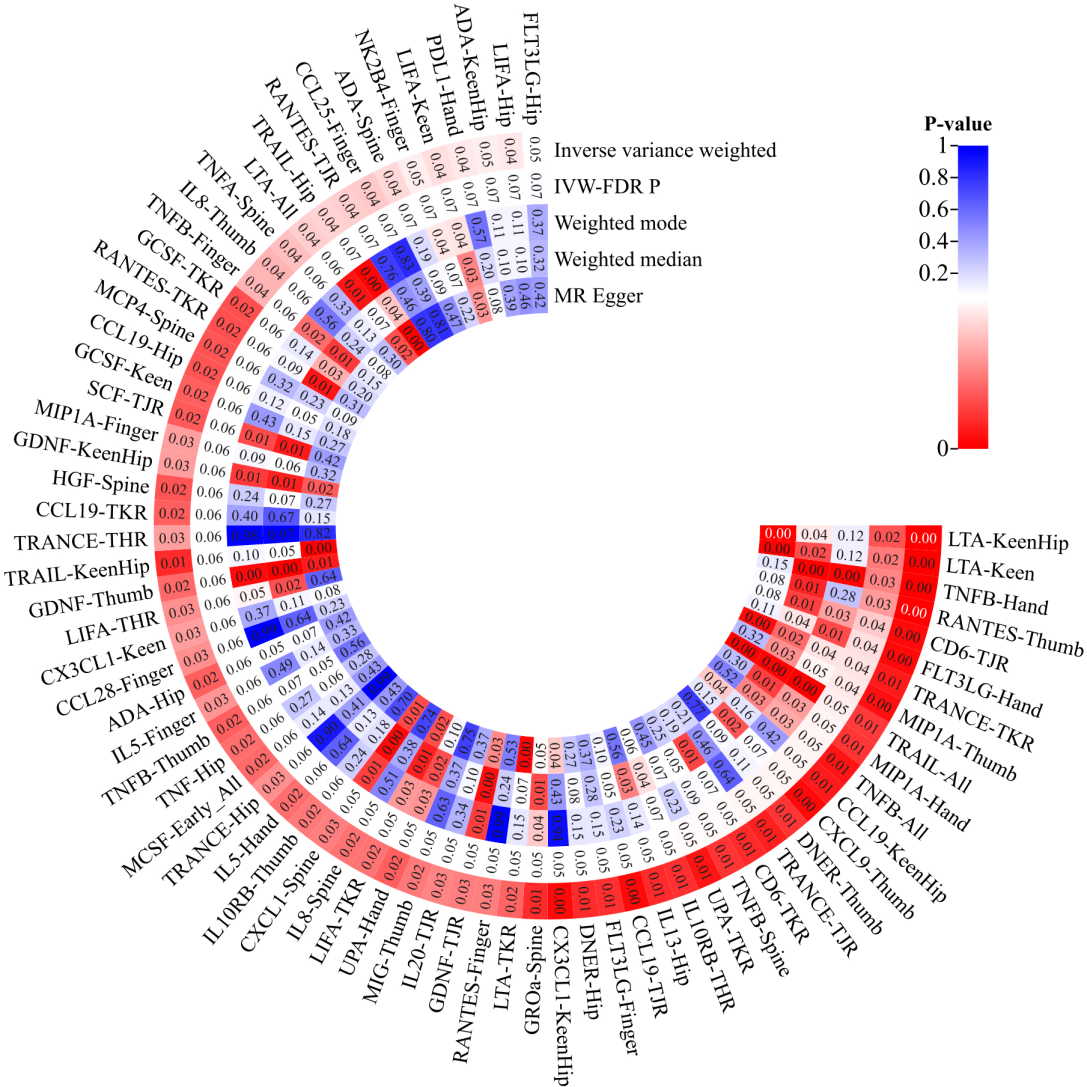
Heatmap of Different MR Analysis Methods for Cytokines and the Risk of Osteoarthritis.

**Figure 3:**
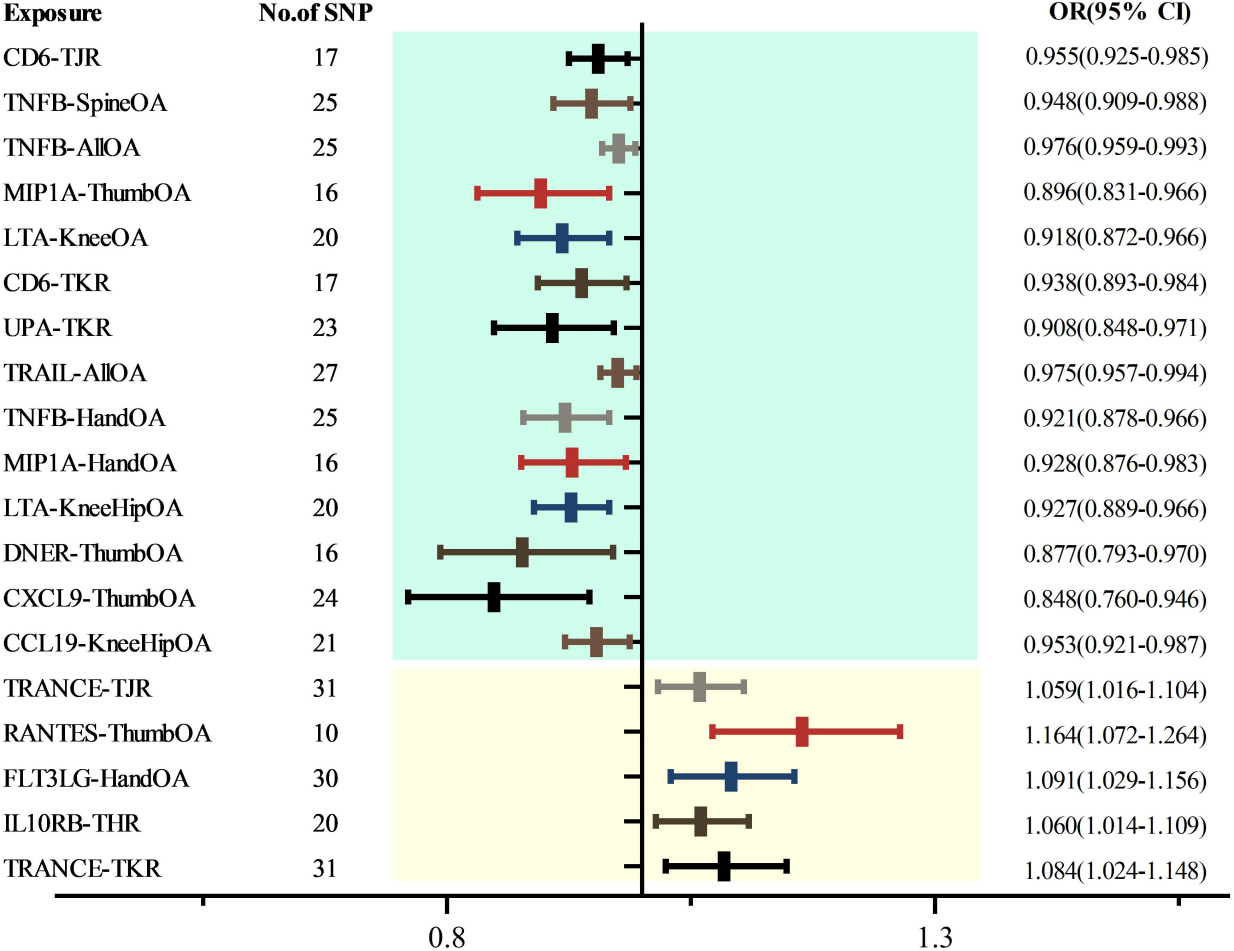
Forest map of cytokines and the risk of osteoarthritis.

Upon eliminating redundant cytokines, the analysis incorporated a total of 103 unique cytokines from diversified sources (**Supplementary Table 2**). Utilizing a significance criterion of P<5×10^-6, the study identified 1677 SNPs associated with the 103 cytokines. These SNPs exhibited an F-statistic range spanning from 18 to 1510, thereby confirming the statistical potency and reliability of the selected cytokines as instrumental variables (IVs) in the MR analysis (**Supplementary Table 3**). In an expansive MR analysis that focused on the 103 cytokines and 12 distinct OA traits, the Inverse Variance Weighted (IVW) method elucidated potential causal associations between 35 cytokines and the 12 specific OA characteristics (**Supplementary Table 4**; **Figure 2**). Post-FDR adjustment, negative correlations with varying OA traits were observed for higher levels of CCL19 [KneeHipOA, 0.953 (0.921-0.987), P=0.047], CD6 [TJR, 0.955 (0.925-0.985), P=0.039; TKR, 0.938 (0.893-0.984), P=0.048], CXCL9 [ThumbOA, 0.848 (0.760-0.946) were found.), P=0.047] DNER [ThumbOA, 0.877 (0.793 0.970), P=0.047], LTA [KneeHipOA, 0.927 (0.889 0.966), P=0.024; KneeOA, 0.918 (0.872 0.966), P=0.017], MIP1A [HandOA, 0.928 (0.876 0.983), P=0.040; ThumbOA, 0.896 (0.831 0.966), P=0.045], TNFB [AllOA.0.976 (0.959 0.993), P=0.047; HandOA, 0.921 (0.821 78-0.966), P=0.026; SpineOA, 0.948 (0.909-0.988), P=0.048], TRAIL [AllOA, 0.975 (0.957-0.994), P=0.046], and UPA [TKR, 0.908 (0.848 0.971), P=0.048]. Conversely, significant positive correlations with OA risk were found for elevated levels of FLT3LG [HandOA, 1.091 (1.029 1.156), P=0.043], IL10RB [THR, 1.060 (1.014 1.109), P=0.049], RANTES [ThumbOA, 1.164 (1.072 1.264), P=0.030], and TRANSCE [TJR, 1.059 (1.016 1.104), P=0.047; TKR, 1.084 (1.024 1.148), A significant correlation was observed between OA risk with diverse characteristics and a P-value of 0.044 (**Figures 2**, **3**). The consistency of MR Egger, Weighted Median, and Weighted Mode methods with the IVW trend in MR analysis underscores the reliability of these results.

In the sensitivity analysis, no SNP pleiotropy was detected in the level pleiotropy and MR-PRESSO analysis (**Supplementary Table 5**), indicating the robustness of our instrumental variables. Additionally, the heterogeneity test revealed no heterogeneity in the MR results (**Supplementary Table 6**), further supporting the reliability of our findings. The LOO method also did not identify any significant bias points (**Supplementary Figure 1**).

In the MR analysis assessing potential causal relationships between 12 OA traits and cytokines, no such relationships were established post-FDR correction (**Supplementary Table 7**). This outcome underscores the complexity of the relationship between OA and cytokines. Overall, our results are reliable and suggest that multiple cytokines may be causally linked to OA.

### 3.2 Replication results of the risk of osteoarthritis

**Figure 4:**
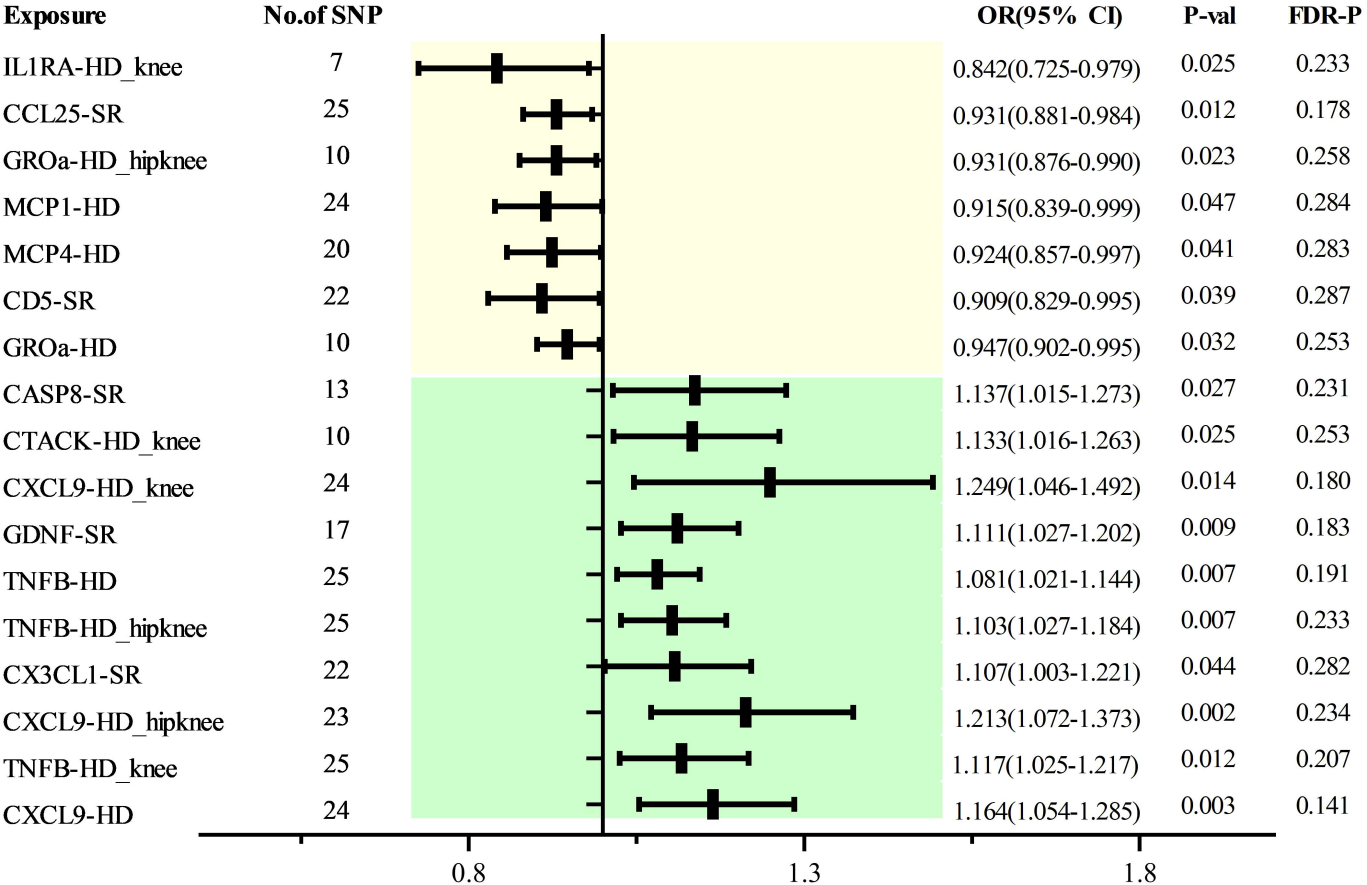
Forest Map of MR Analysis of Cytokines and Risk of Osteoarthritis (replication).

As with discovery analysis, we extracted a GWAS data of 5 OA traits from the database, including 1649 sufficiently robust SPNs (F>10) related to 103 cytokines (**Supplementary Tables 1**, **8**). MR analysis found that CD5 [SR, 0.909 (0.829 0.995), P=0.039], CCL25 [SR, 0.931 (0.881 0.984), P=0.012], IL0RA [HD_ken, 0.842 (0.725 0.979), P=0.025], GROa [HD_hipkeen, 0.931 (0.876 0.990), P=0.023], MCP1 [HD, 0.915 (0.893 0.999), P=0.047], MCP4 [HD, 0.924 (0.857 0.997), P=0.041] were associated with different features of OAA. There is a causal relationship, while CASP8 [SR, 1.137 (1.015-1.273), P=0.027], CATCK [HD_ken, 1.133 (1.016-1.263), P=0.025] CXCL9 [HD_hipkeen, 1.249 (1.046 1.492), P=0.014], GDNF [SR, 1.111 (1.027 1.202), P=0.009], TNFB [HD, 1.081 (1.021 1.144), P=0.007; HD_hipkeen, 1.103 (1.027 1.184), P=0.007; HD_ken, 1.117 (1.025 1.217), P=0.012], CX3CL1 [SR, 1.107 (1.003 1.224), P=0.044], and CXCL9 [HD_hipkeen, 1.213 (1.072 1.373), P=0.002; HD, 1.164 (1.054 1.285), P=0.003] indicated increased OA risk. However, post-correction, no significant causal relationships were established among all cytokines (**Figure 4**). Horizontal pleiotropy testing and MR-PRESSO did not show heterogeneity or pleiotropy (**Supplementary Tables 9**, **10**), and the homogeneity test of MR results also indicated no heterogeneity (**Supplementary Table 11**). LOO analysis further confirmed the robustness of these findings (**Supplementary Figure 2**). Reverse MR analysis did not demonstrate causal relationships between different OA traits and the aforementioned cytokines (**Supplementary Table 12**).

### 3.3 Combined results of osteoarthritis risk from the meta-analysis

**Figure 5:**
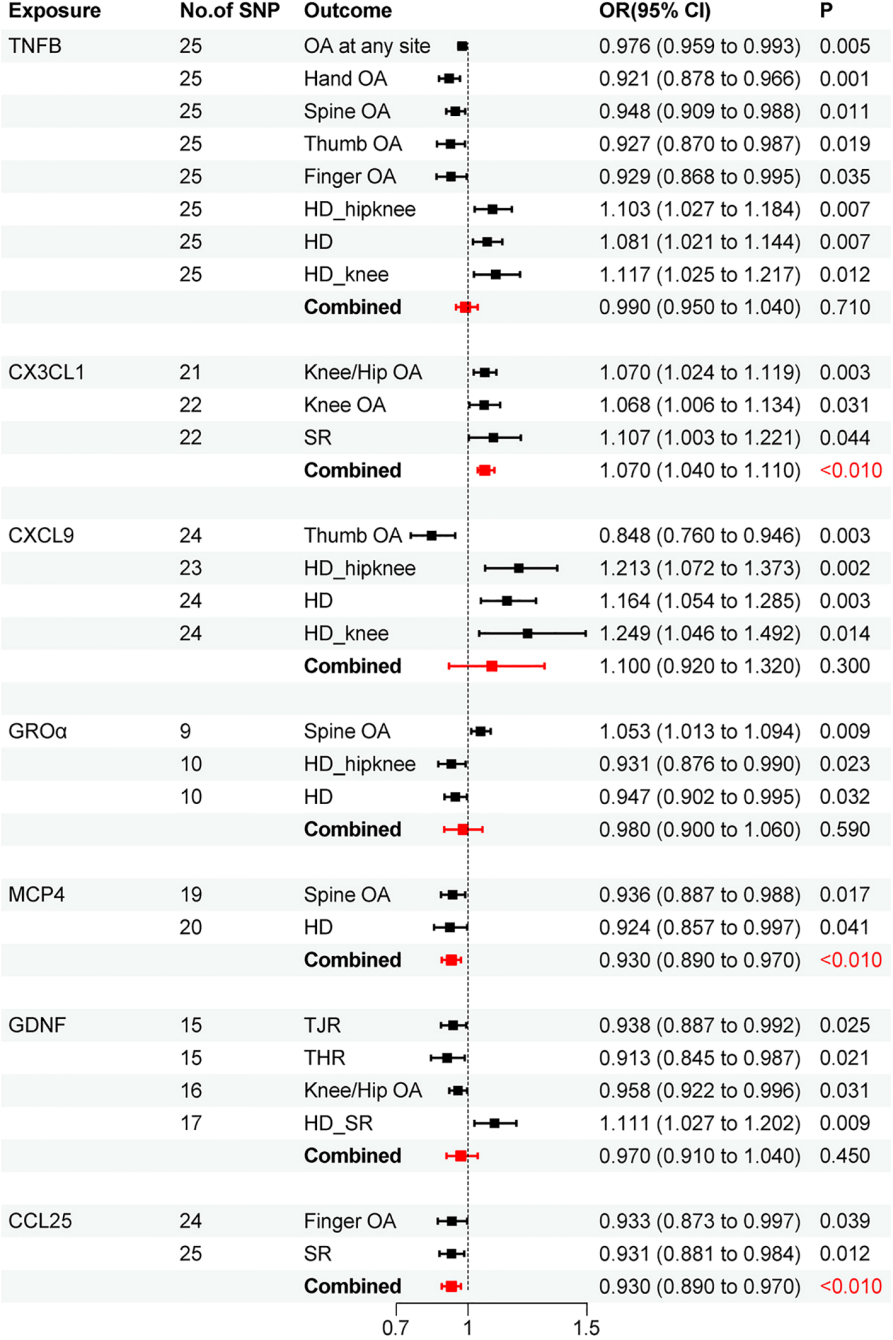
Forest Map of combined results of osteoarthritis risk from the meta-analysis.

Through meta-analysis, we ultimately found a positive causal relationship between CX3CL1 and OA (OR=1.070,95% CI: 1.040-1.110; P<0.010), while MCP4 (OR=0.930,95% CI: 0.890-0.970; P<0.010) and CCL25 (OR=0.930,95% CI: 0.890-0.970; P<0.010) demonstrated negative causal relationships with OA (**Figure 5**).

## 4 Discussion

OA is a highly complex degenerative disease, with its pathogenesis being multifaceted and intricate. Increasing evidence underscores the significance of cytokines in the onset and progression of OA. However, the causal link and mechanism of action between cytokines and OA remain largely undefined. Our study utilized the most comprehensive set of cytokine type SNPs with identified sites and the largest OA GWAS database for discovery MR, supplemented by ample OA GWAS data for replication MR, to assess the causal impact of cytokines on OA. Collectively, the integration of MR analysis and meta-analysis highlighted a causal relationship between three distinct cytokines and OA.

Research indicates significant correlations between CCL19, CD6, CXCL9, DNER, LTA, MIP1A, TNFB, TRAIL, UPA, FLT3LG, IL10RB, RANTES, TRANSCE, and OA or bone metabolism (28–40). Our genetic MR analysis corroborated the causal association between these cytokines and OA. The meta-analysis integrated evidence suggesting a causal link between elevated levels of CCL25 and MCP4 and reduced OA risk. Conversely, an increase in CX3CL1 levels correlates with heightened OA risk.

CCL25 (TECK) is a key small molecular weight chemokine and a vital component of the inflammatory system. Typically, CCL25 interacts with its receptor CCR9, contributing to cell migration and metastasis, and it plays a role in recruiting inflammatory factors, thereby enhancing the inflammatory response. Moreover, CCL25 is involved in the chemotaxis of T lymphocytes, participating in a variety of physiological and pathological processes, including immune cell development, differentiation, and allergic diseases. It is recognized as a surface marker of homing lymphocytes (41). However, the relationship between CCL25 and OA has not been extensively reported. In research on porcine mesenchymal stem cells (MSCs) and their migration and chondrogenesis induced by CCL25, it was observed that CCL25 notably influenced MSC chemotaxis without causing significant cell death, even at higher concentrations. Studies suggest that certain concentrations of CCL25 may promote cartilage repair (42). In experiments involving intra-articular CCL25 injections in guinea pig knee OA, CCL25 not only effectively delayed OA progression and reduced cartilage damage (as indicated by lower Mankin scores) but also significantly increased MSC migration, suggesting that high CCL25 levels might be beneficial in preventing or treating OA (43). Additionally, a study on the chemotactic potential of CCL25 on human mesenchymal progenitor cells from subchondral cortical sponge bone in normal, rheumatoid arthritis (RA), and OA synovial fluid (SF) showed that CCL25 could enhance subchondral progenitor cell migration and induce cartilage tissue repair (44). Our genetic MR analysis investigated the causal relationship between CCL25 and OA, aligning with these findings. The results indicate that CCL25 may exert a protective effect against OA. However, reverse MR analysis did not establish a direct causal link between OA and CCL25.

MCP-4 is a chemokine known for its affinity to CCR3, and it has been implicated in autoimmune diseases like RA as well as allergic conditions (45, 46). It mediates the migration of eosinophils, basophils, macrophages, and lymphocytes in allergic diseases and possesses marked chemotactic activity for monocytes and T lymphocytes in inflammatory contexts (47). To date, no studies explicitly delineate the role of MCP-4 in OA. However, a study on MCP-4 expression in cartilage tissue, using OA and normal human articular cartilage as controls, revealed that MCP-4 expression was significantly elevated in OA cartilage compared to the normative cohort (48). Additionally, body mass index (BMI), known to be positively correlated with OA risk, has been linked to MCP-4. A Japanese study involving 39 overweight individuals showed that serum MCP-4 levels positively correlated with BMI, waist circumference, waist-to-hip ratio, and hypersensitive C-reactive protein (49). Another study focusing on severely obese patients confirmed that MCP-4 serum levels were elevated compared to those in normal-weight individuals and positively correlated with BMI (50). Our MR analysis indicates a causal relationship between elevated MCP-4 expression and reduced OA risk. However, the connection between MCP4 and OA pathogenesis remains unexplored. Our MR results suggest a causal link between high MCP4 expression and a reduced risk of OA. Yet, reverse MR did not identify a causal relationship between OA and MCP4. Given these conflicting findings, further research, particularly focusing on underlying mechanisms, is essential to clarify MCP4’s potential role in OA development.

CX3CL1, the sole member of the CX3C class of chemokines, exerts chemotactic effects on T cells and monocytes and is involved in various signaling pathways, such as p38MAPK and Akt. These pathways play a crucial role in mediating inflammatory diseases (51–53). Studies focusing on OA and related pain behaviors in mice have demonstrated that CX3CL1 is intricately linked to OA and varying pain intensities (54). In research on degenerative joint disease-related pain in cats, CX3CL1 levels were found to be upregulated in both the dorsal root ganglia affected by the disease and the spinal cord in cats displaying clinical signs of OA pain, compared to those without (55). A study examining the methylation genes in articular cartilage from OA patients identified CX3CL1 as a differentially elevated gene. Notably, its expression level increased exclusively in the OA group, distinguishing it from the control and RA groups (56), suggesting a potential genetic association with OA. Furthermore, an investigation into the expression levels of CX3CL1 in the serum and synovial fluid of 193 knee OA patients revealed not only elevated levels in OA patients but also a positive correlation with the degree of physical pain and disability (57). Our MR analysis, backed by a sufficient number of SNPs, indicates a positive causal relationship between CX3CL1 and OA. Additionally, the study design effectively excluded the influence of horizontal pleiotropy, reverse causal relationships, and confounding factors.

Previously, MR studies investigating the causal relationship between cytokines and OA identified links with MIP-1B, TNFB, and RANTS (58), aligning with our MR analysis results. Another study found a causal relationship between MCSF and VEGF and OA (19),yet these associations were not replicated in our MR and replication MR analyses. Our study, with its extensive OA sample size and the broadest range of cytokines to date in GWAS, also included discovery MR analysis, replication MR analysis, and meta-analysis, ensuring robust results. Notably, our analysis revealed a causal relationship between MCP4 and OA, a finding unprecedented in existing literature. This novel association warrants further investigation to validate MCP4 as a potential biomarker for OA prevention and treatment.

Despite these advancements, our research has limitations. First, the replication analysis for OA with 12 traits did not yield sufficient OA GWAS features. Additionally, akin to other studies (59–61), the paucity of SNPs led us to set a P-value threshold of <5×10^-6, potentially introducing false positives. However, the F-statistic for SNPs IV in our MR analysis exceeded 10, attesting to the robustness of our results. Finally, while we identified three cytokines potentially causally related to OA, their roles in affecting OA survival or disease progression merit further investigation.

## 5 Conclusion

Our MR analysis, with ample samples and a comprehensive range of cytokines, uncovered a potential causal relationship between CX3CL1, MCP4, and CCL25 and OA risk alterations. These findings, particularly regarding the novel cytokine markers, necessitate additional research to elucidate their mechanisms. The insights gained may significantly contribute to OA prevention and treatment strategies.

## Data availability statement

The original contributions presented in the study are included in the article/**Supplementary Material**. Further inquiries can be directed to the corresponding author/s.

## Author contributions

ZoJ: Conceptualization, Funding acquisition, Methodology, Writing – original draft. XC: Data curation, Methodology, Software, Writing – review & editing. XiY: Formal analysis, Resources, Visualization, Writing – review & editing. SZ: Formal analysis, Supervision, Writing – review & editing. WL: Data curation, Validation, Visualization, Writing – review & editing. ZeJ: Formal analysis, Methodology, Software, Visualization, Writing – review & editing. FT: Project administration, Resources, Supervision, Writing – review & editing. WM: Funding acquisition, Resources, Supervision, Validation, Writing – review & editing. XuY: Funding acquisition, Supervision, Visualization, Writing – review & editing. CC: Investigation, Supervision, Writing – review & editing. TL: Investigation, Supervision, Writing – review & editing.

## Supplementary material

Supplementary Figure 1 Discovery of Leave One Out.

Supplementary Figure 2 Replication of Leave One Out.
