# Supplementary figures and images for: The causal effect of cytokine cycling levels on osteoarthritis: a bidirectional Mendelian randomized study
Source: Front Immunol. 2024 Jan 11;14:1334361. doi: 10.3389/fimmu.2023.1334361 (PMC10808687; doi:10.3389/fimmu.2023.1334361)

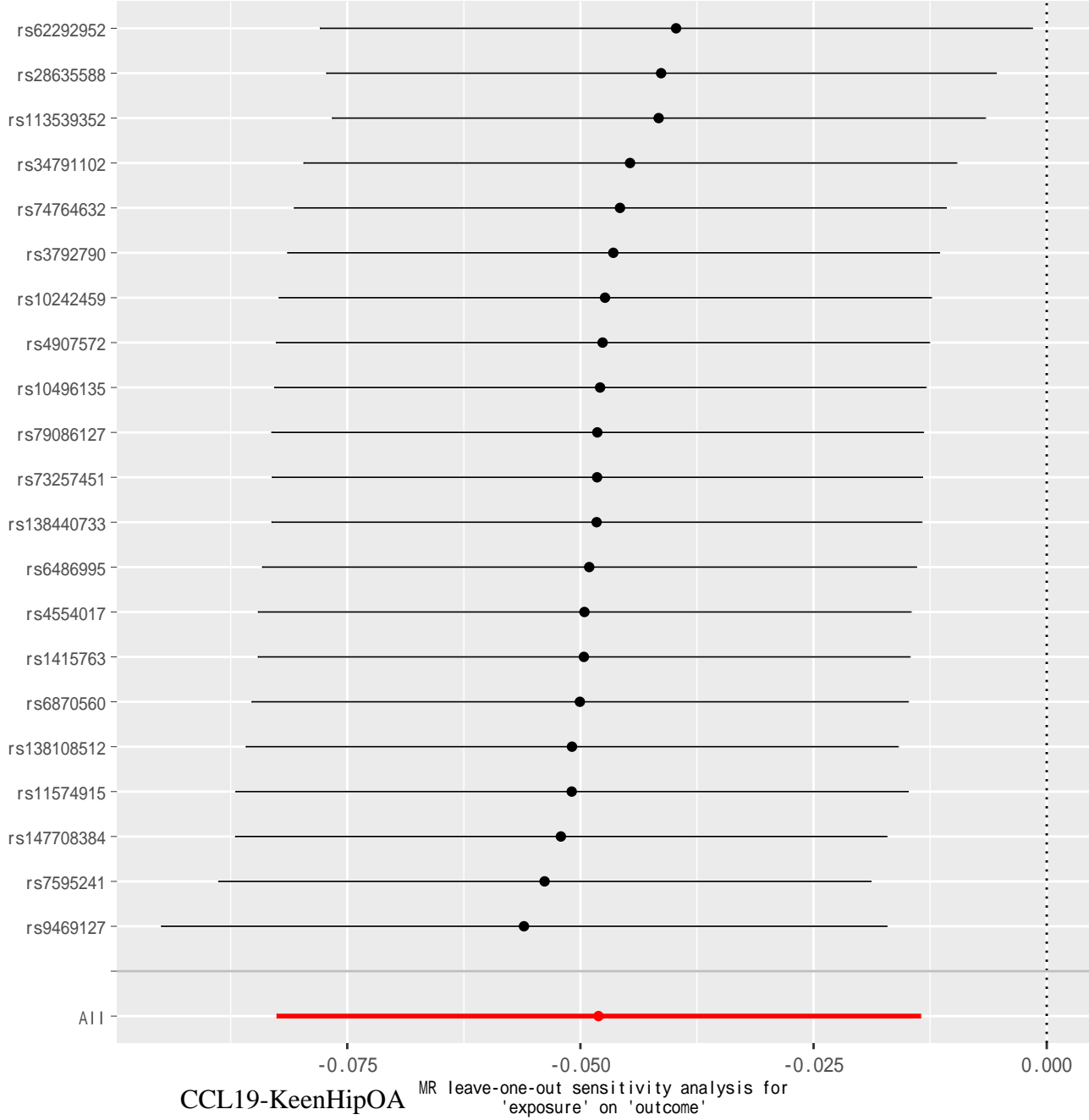

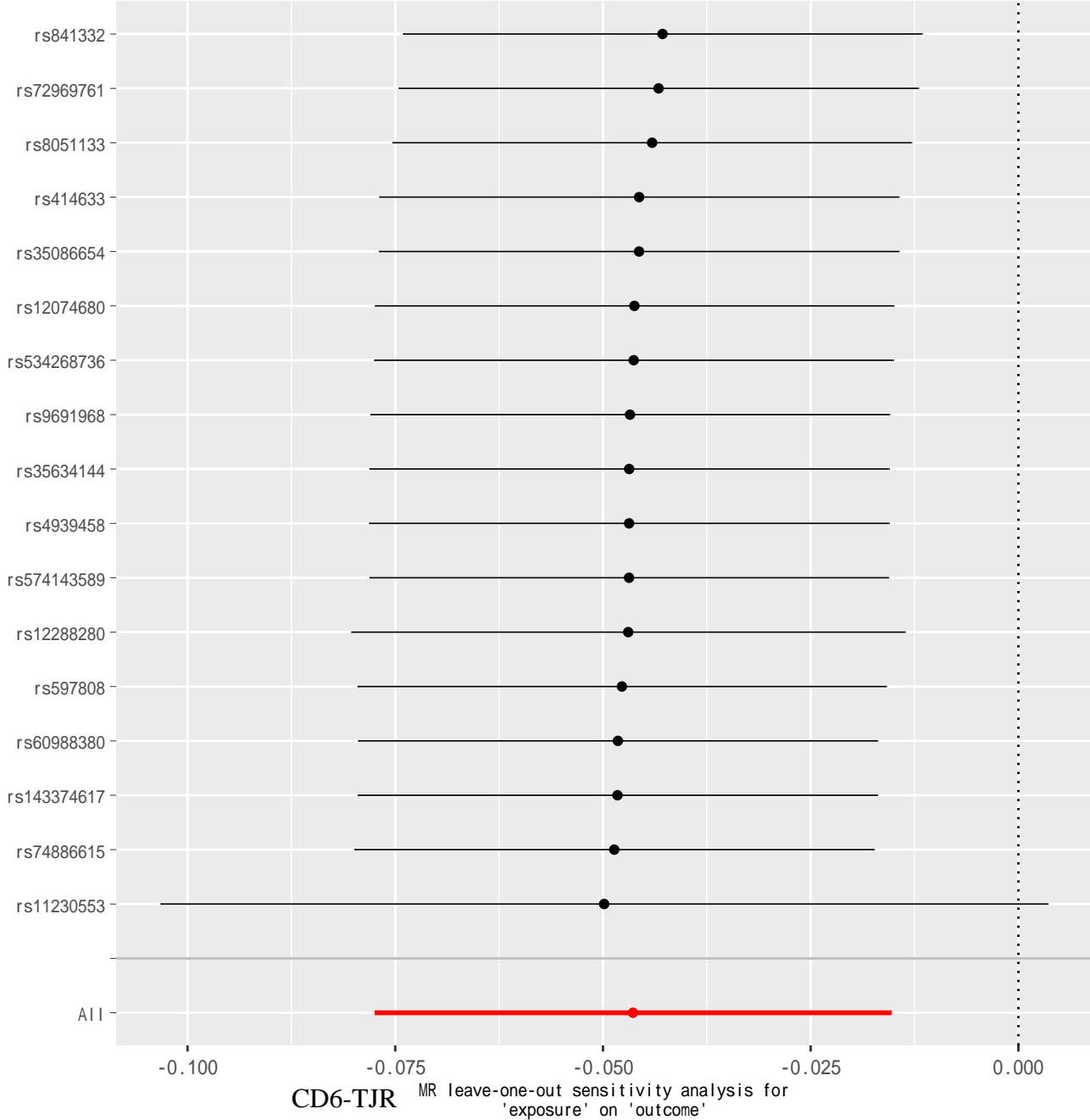

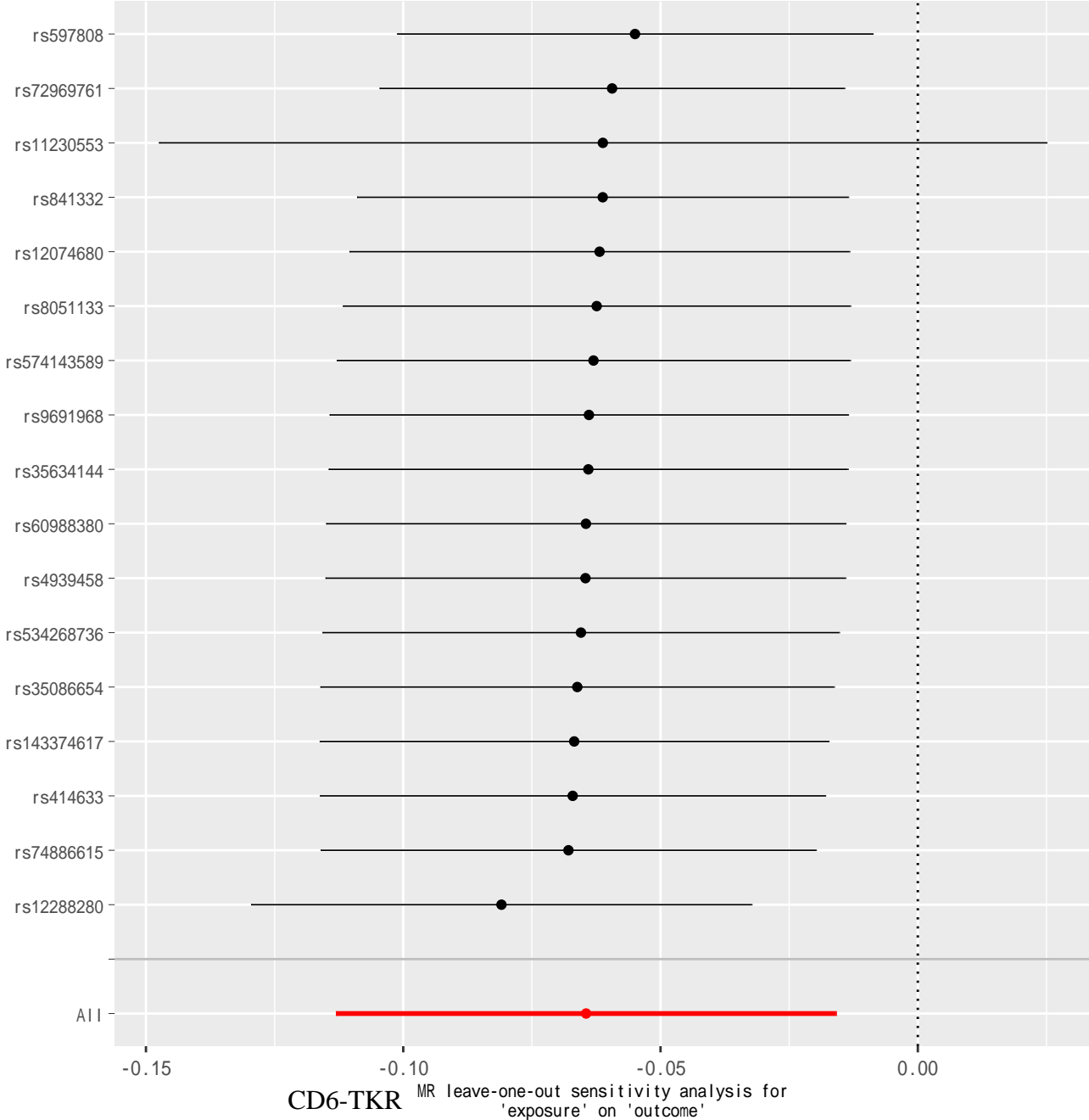

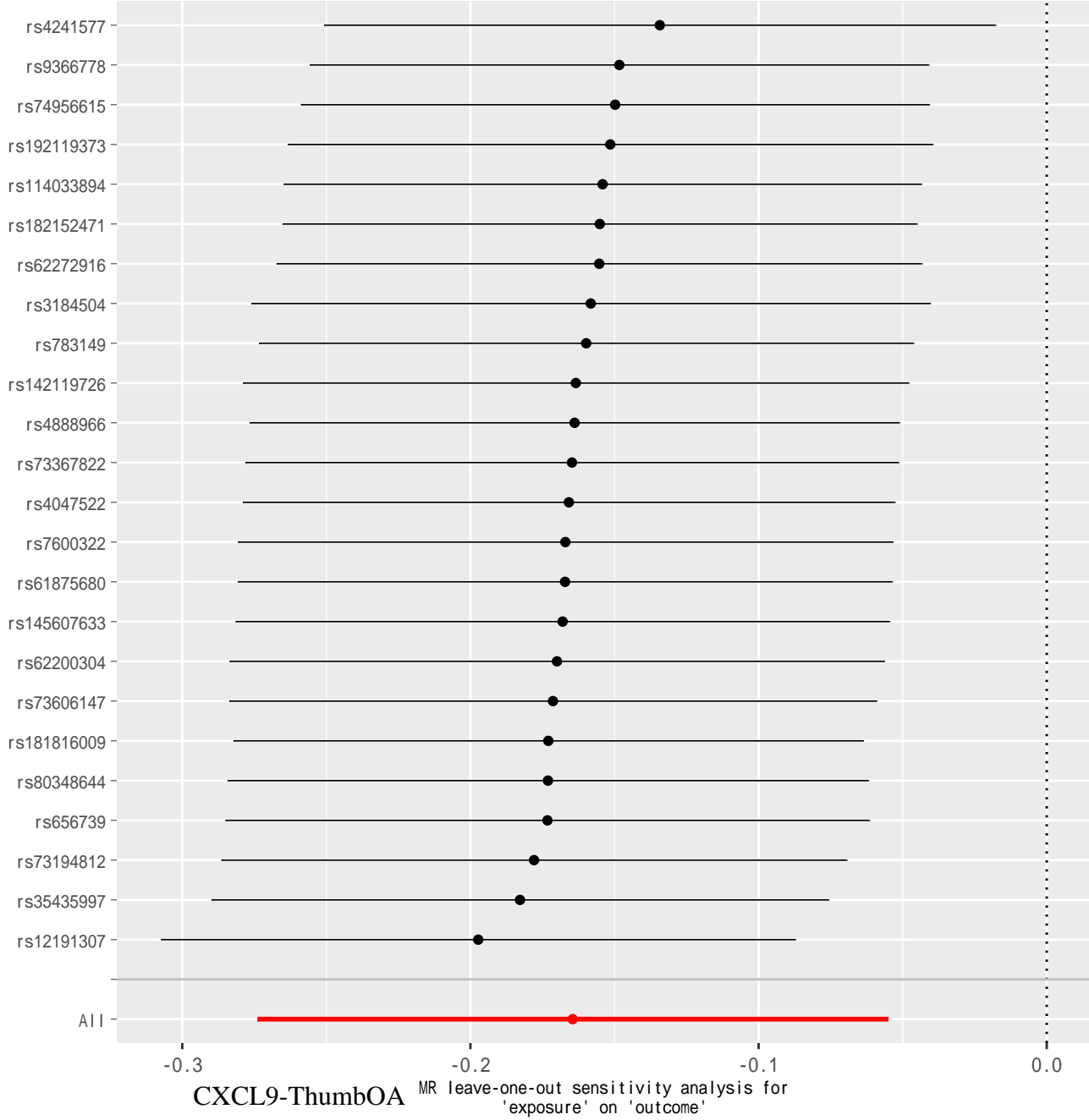

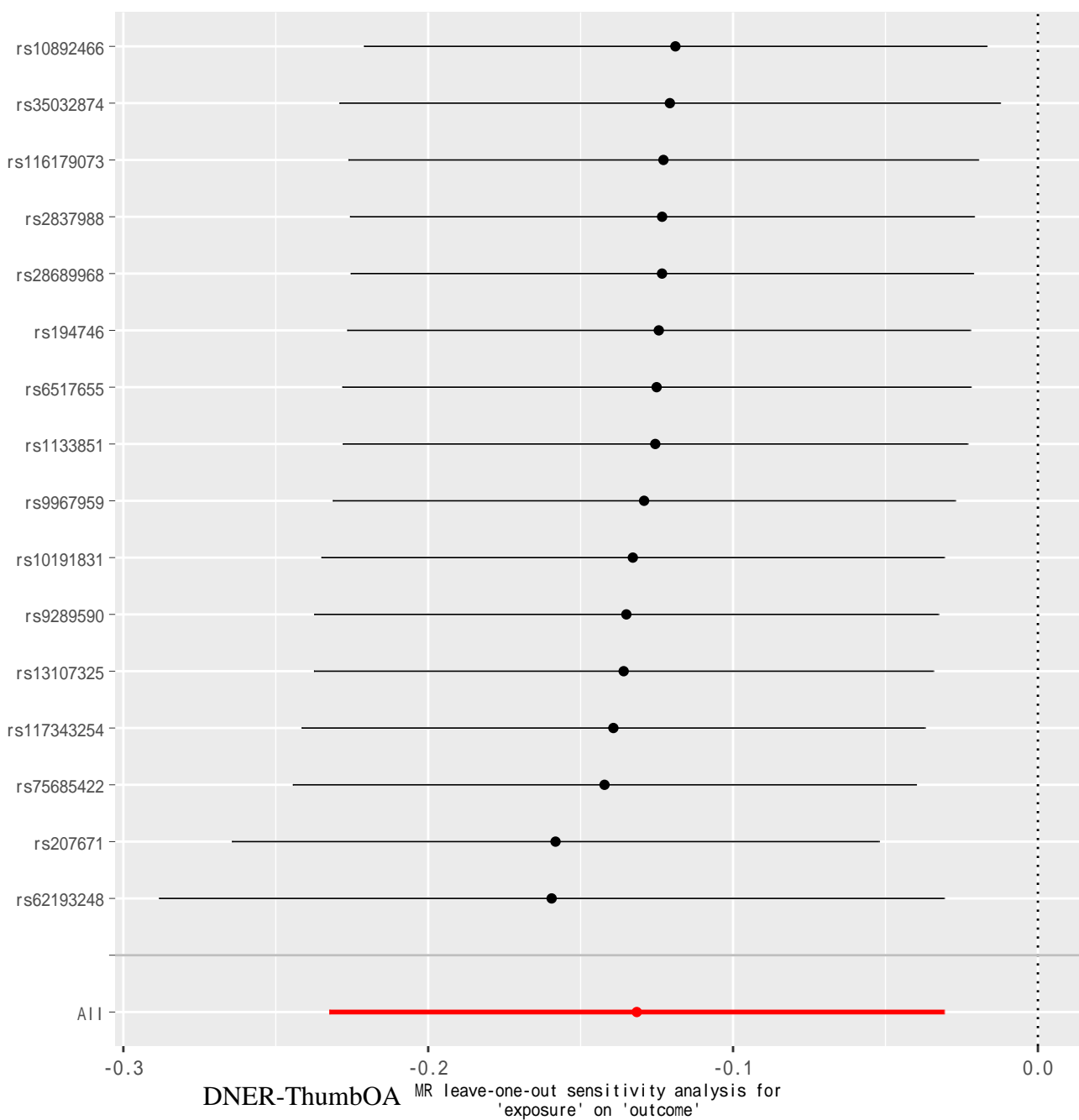

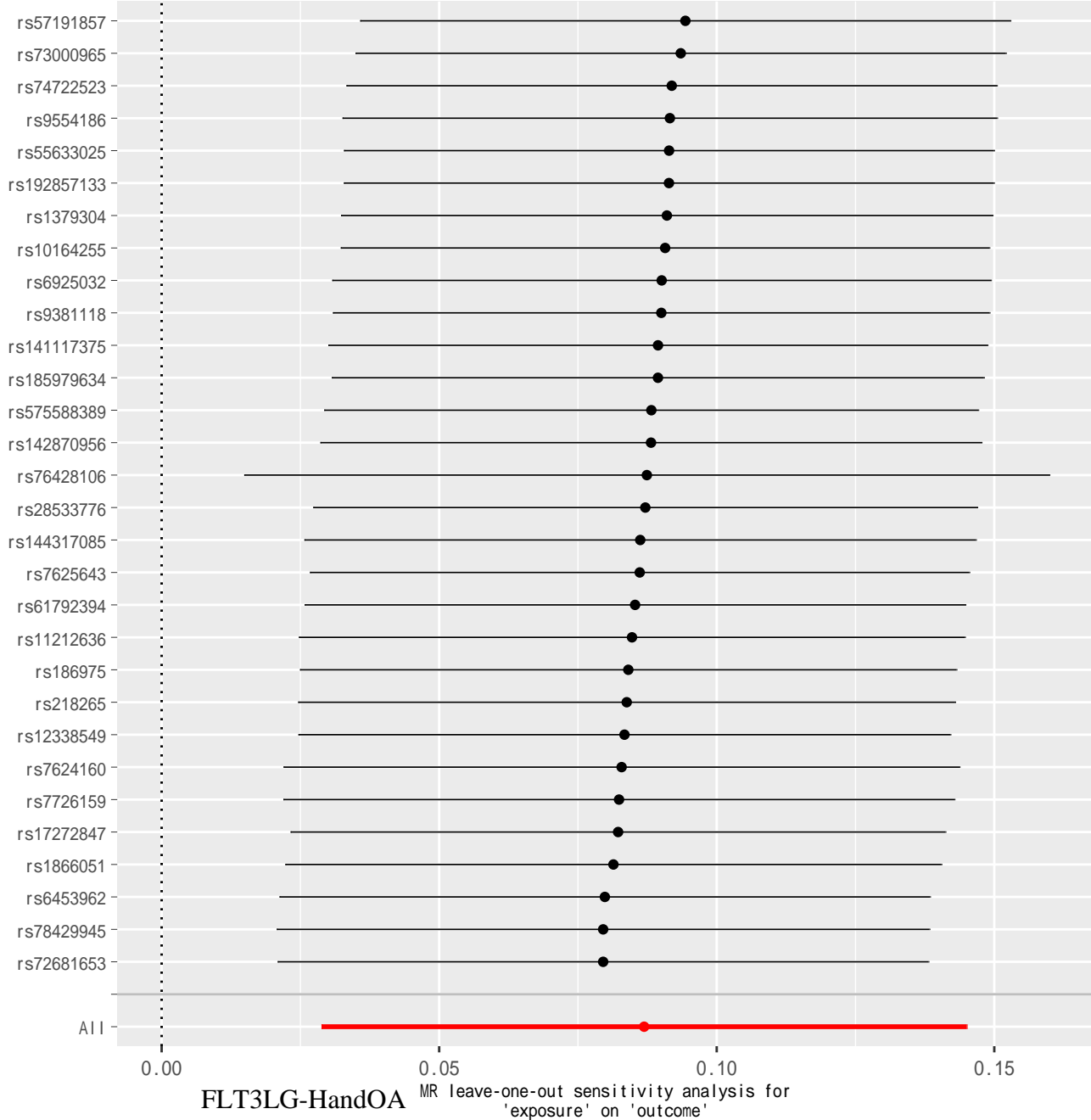

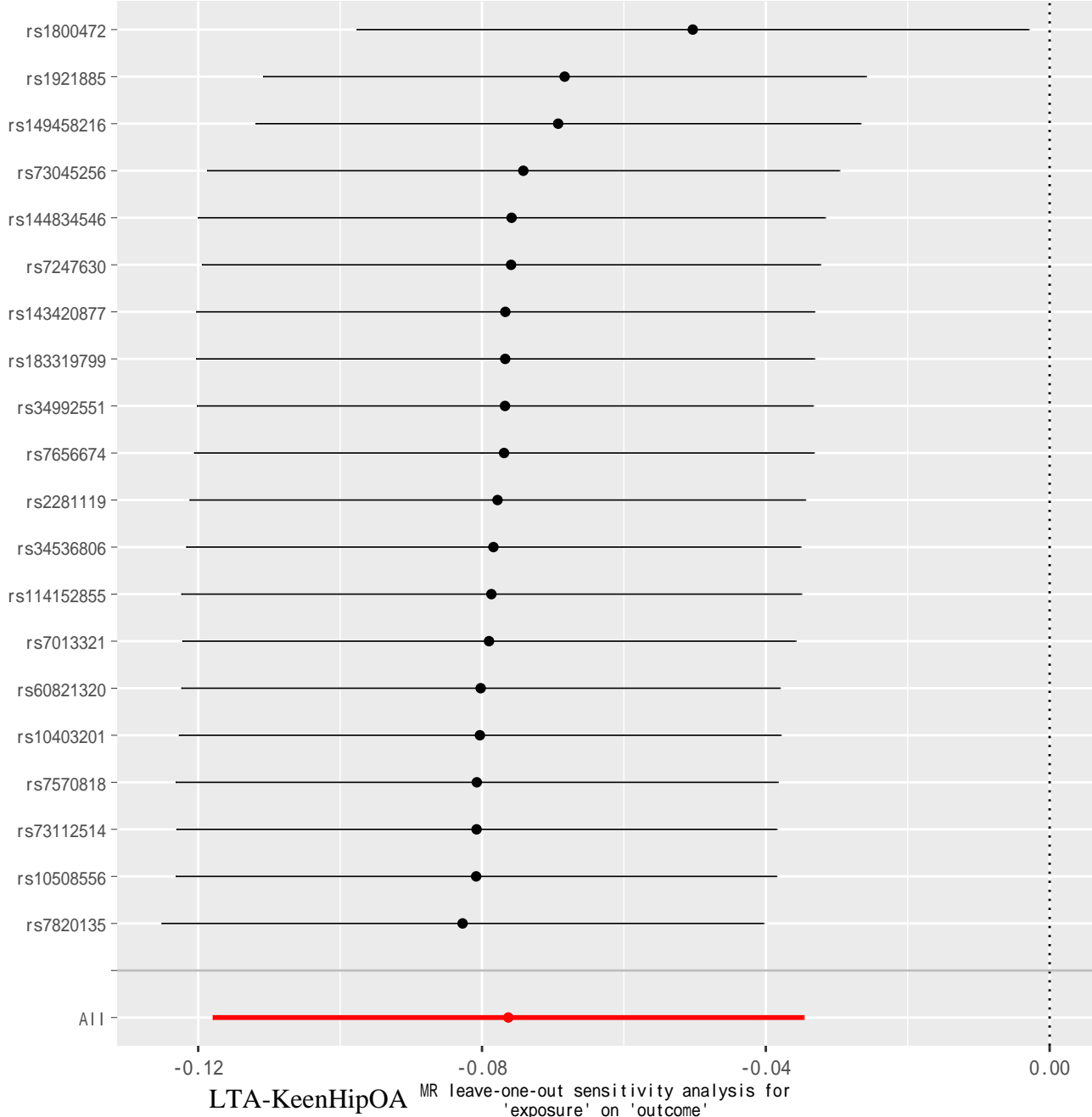

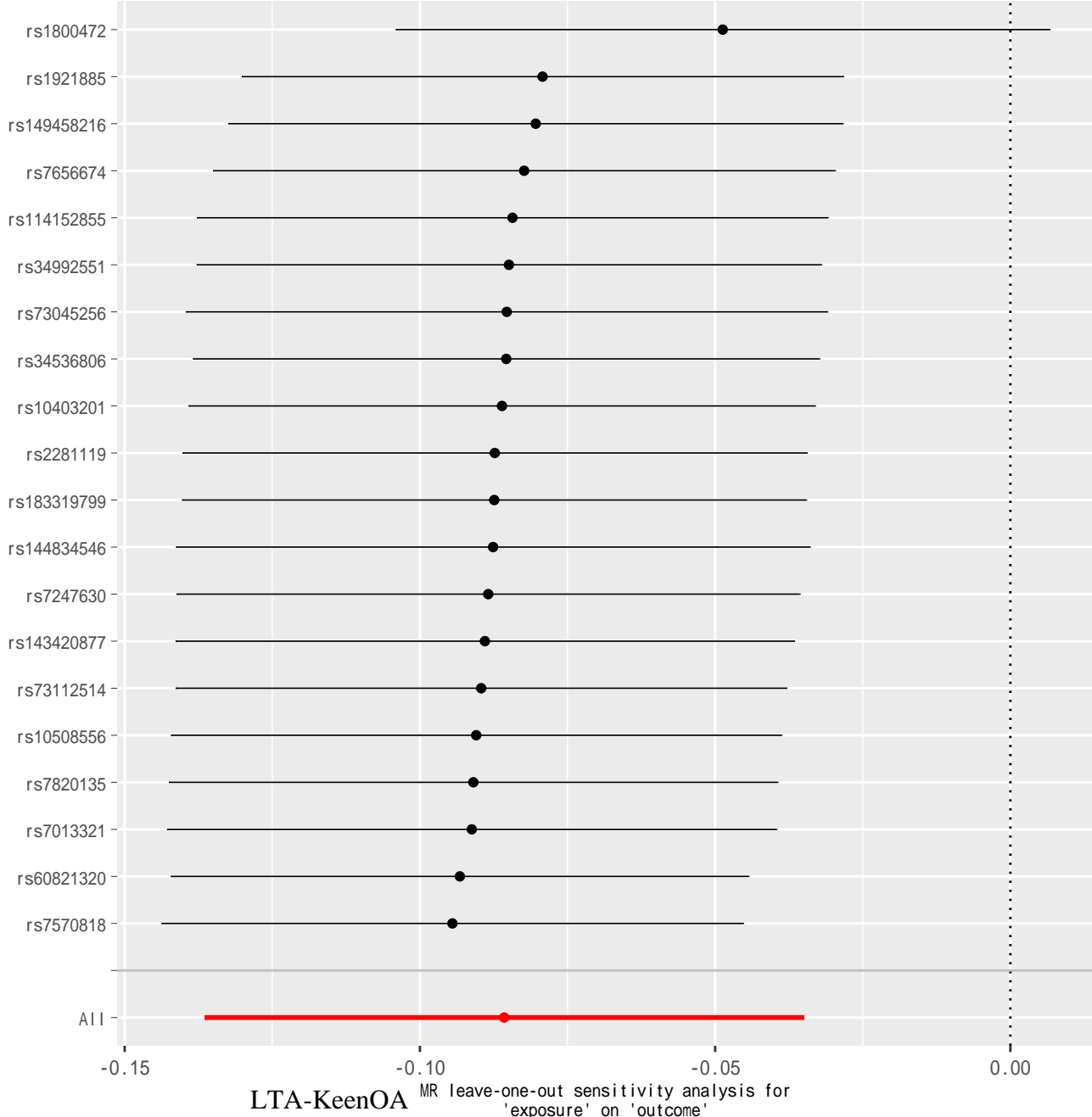

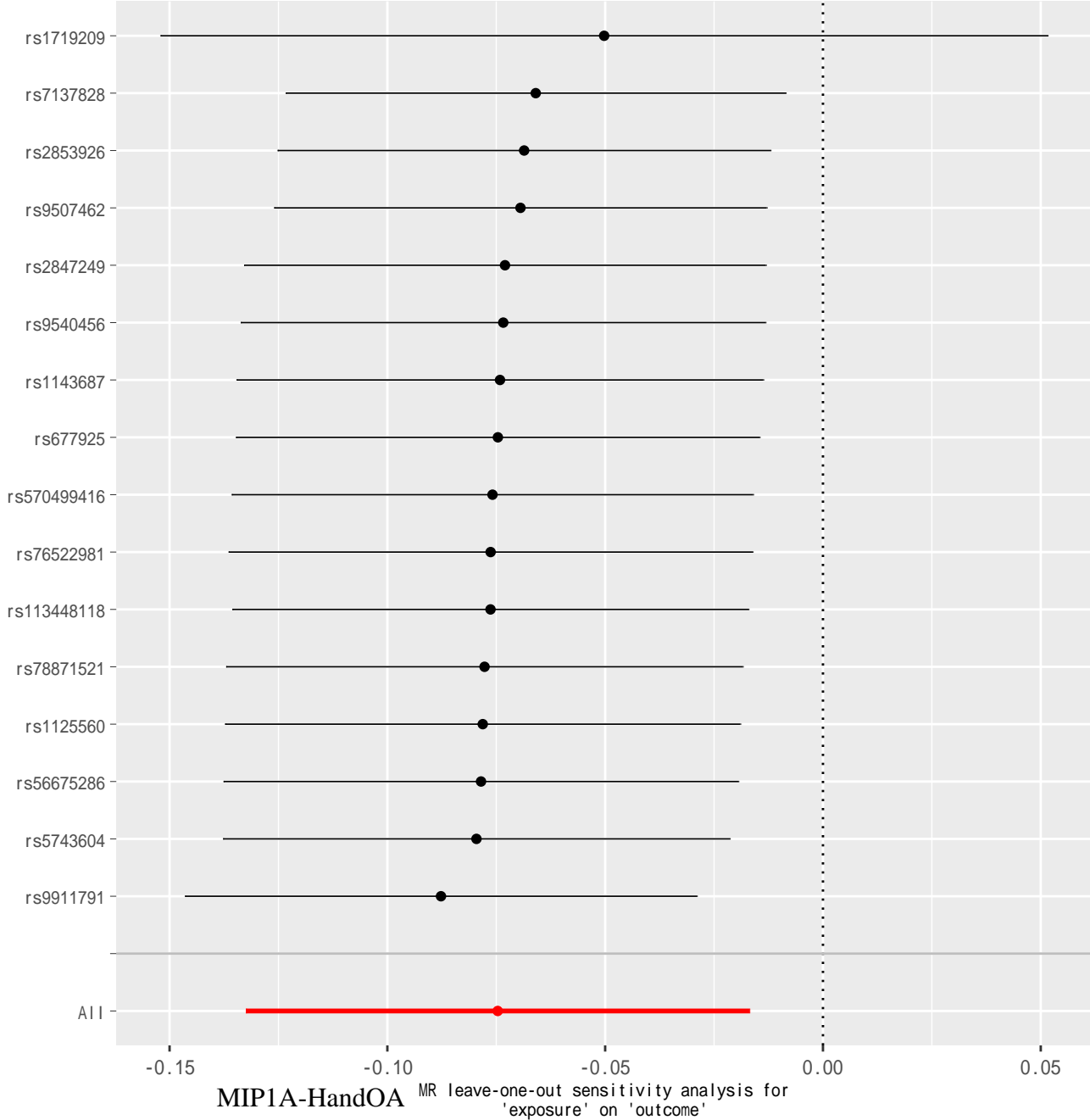

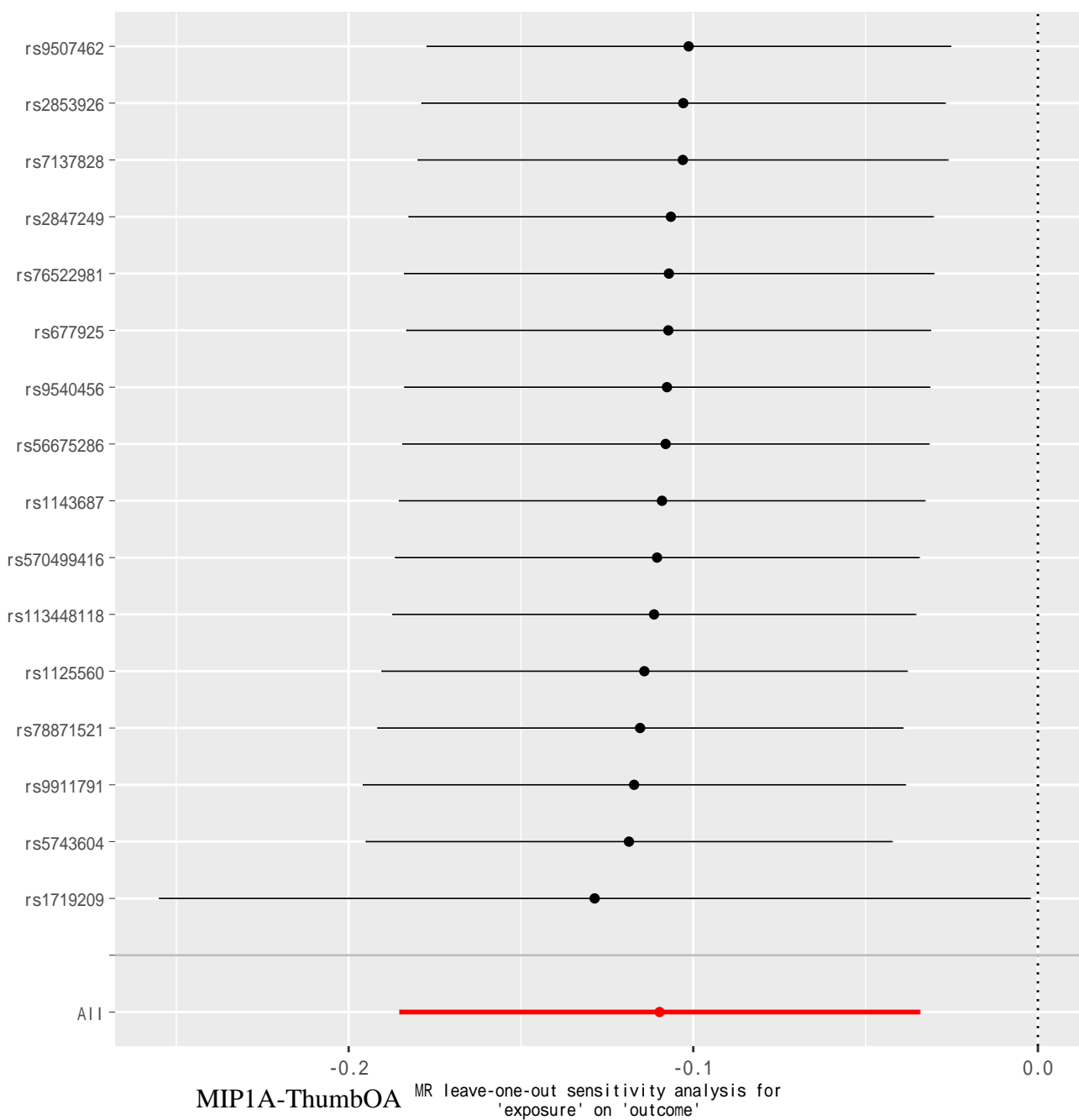

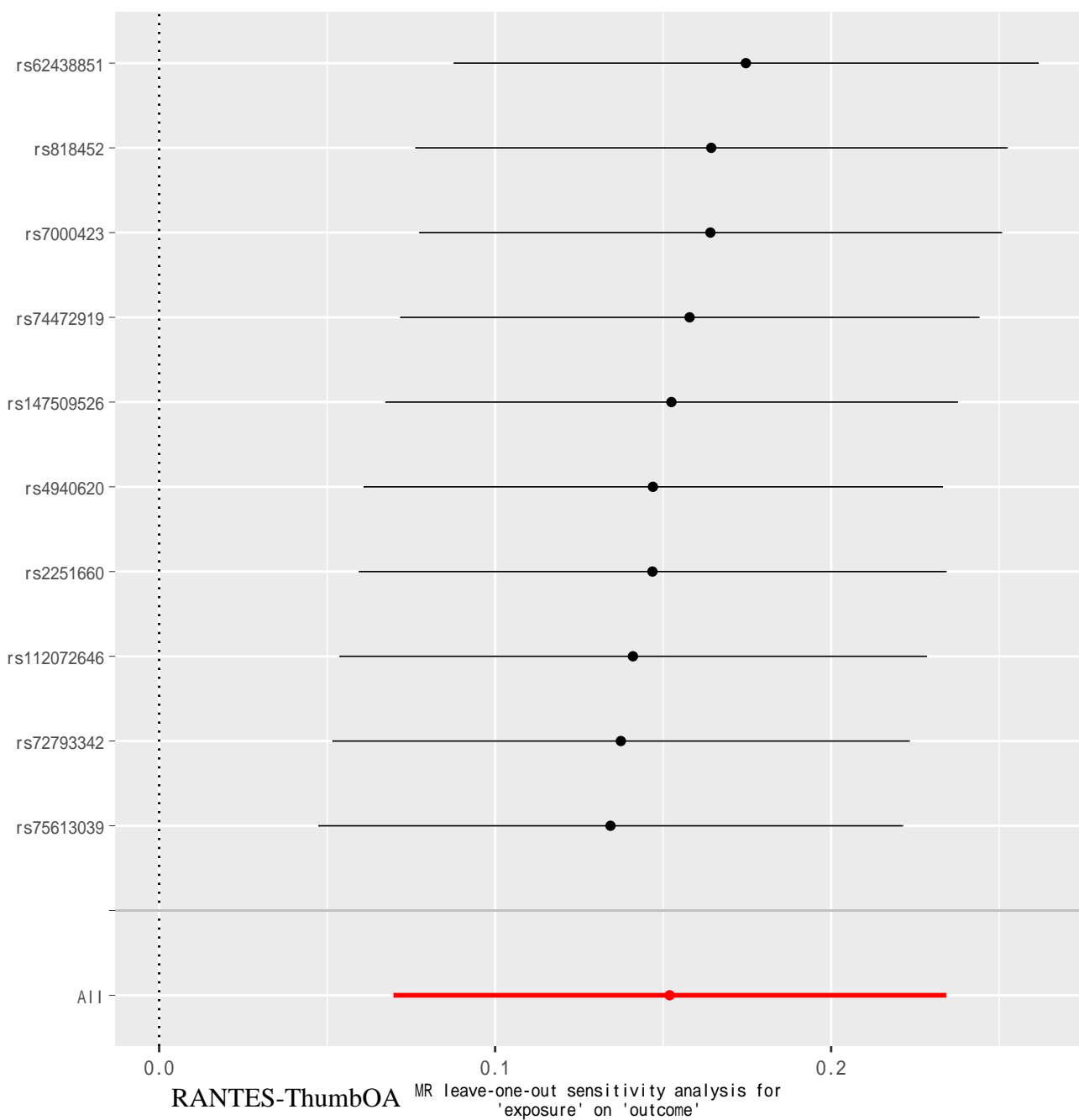

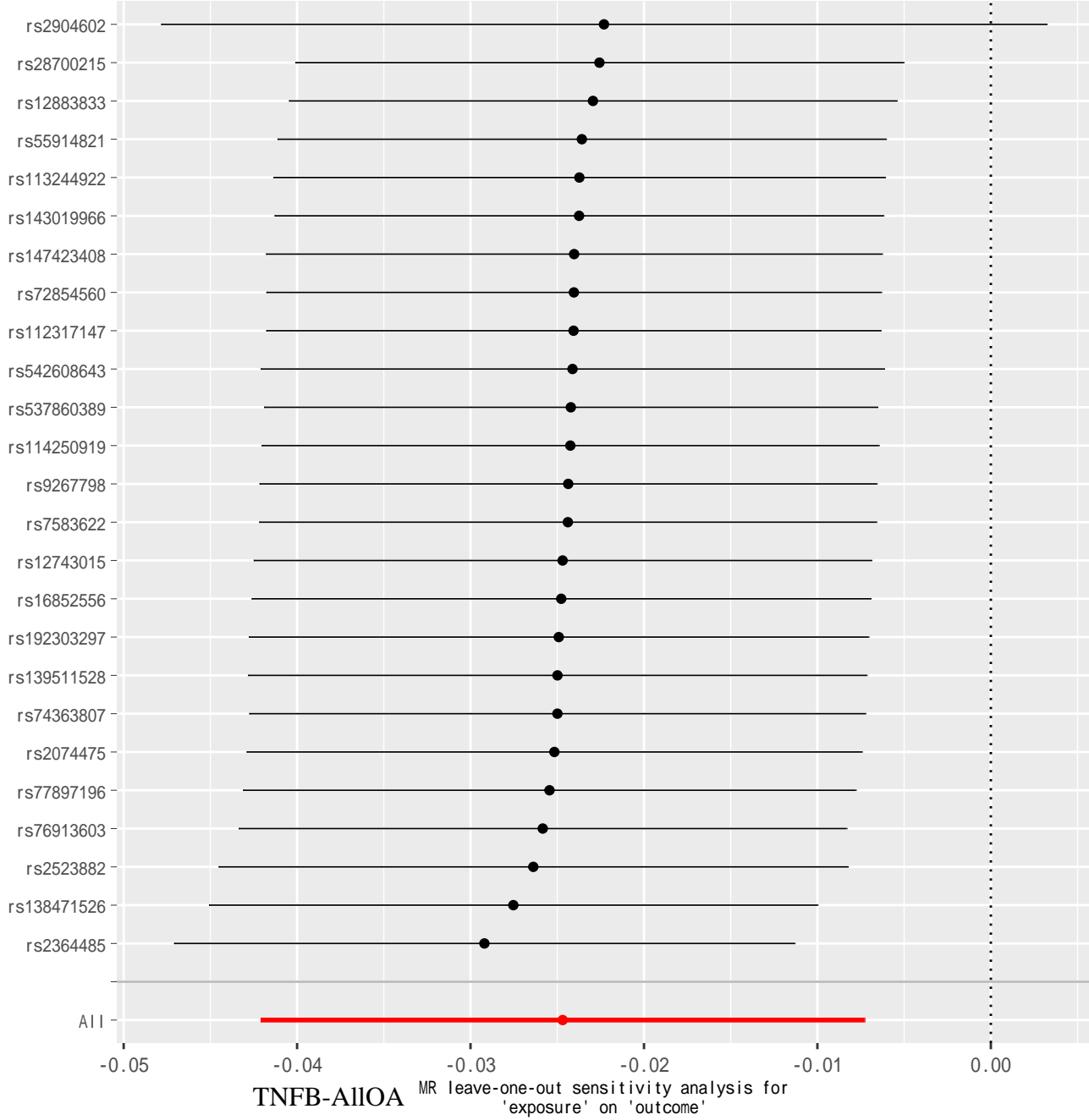

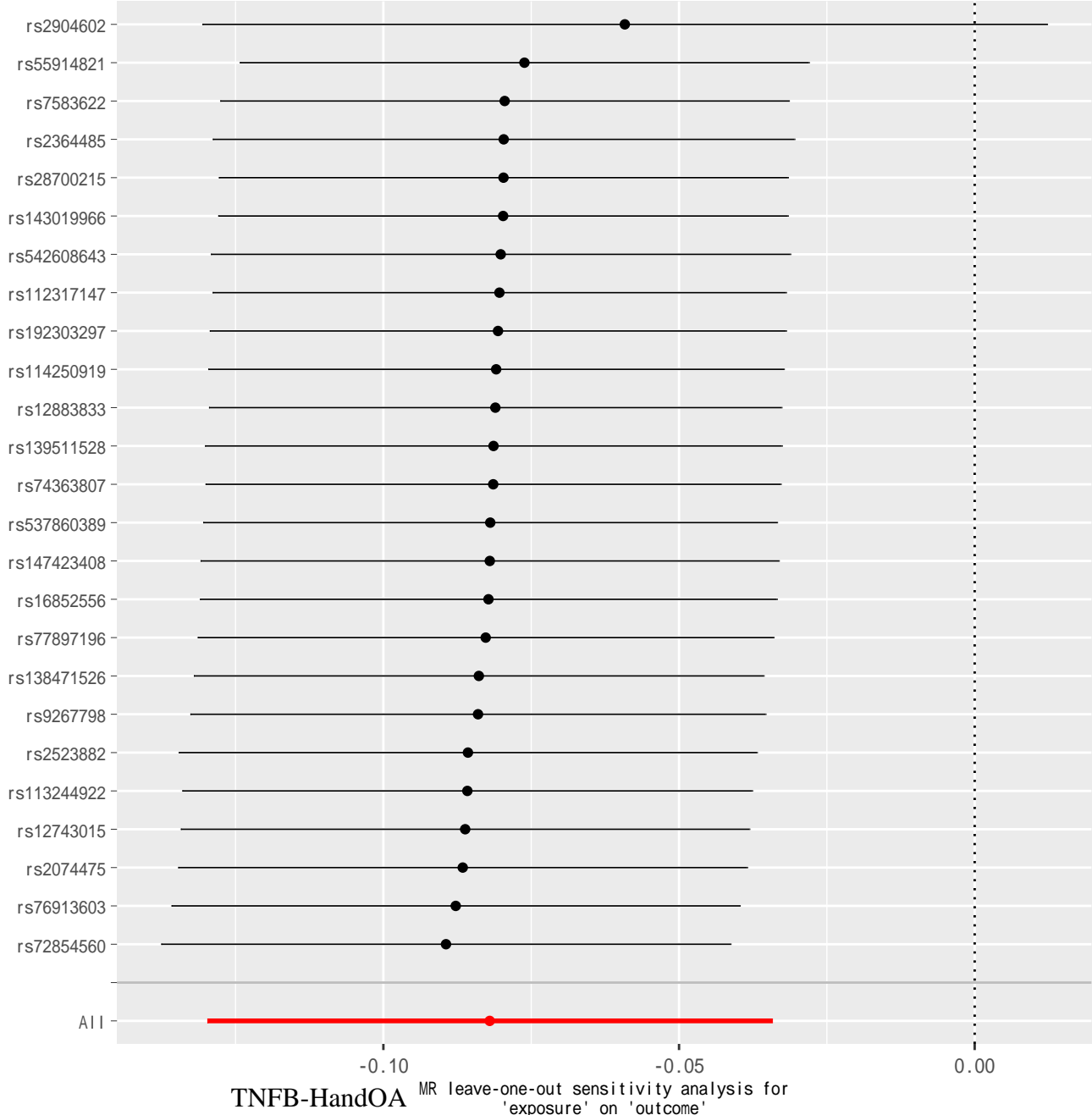

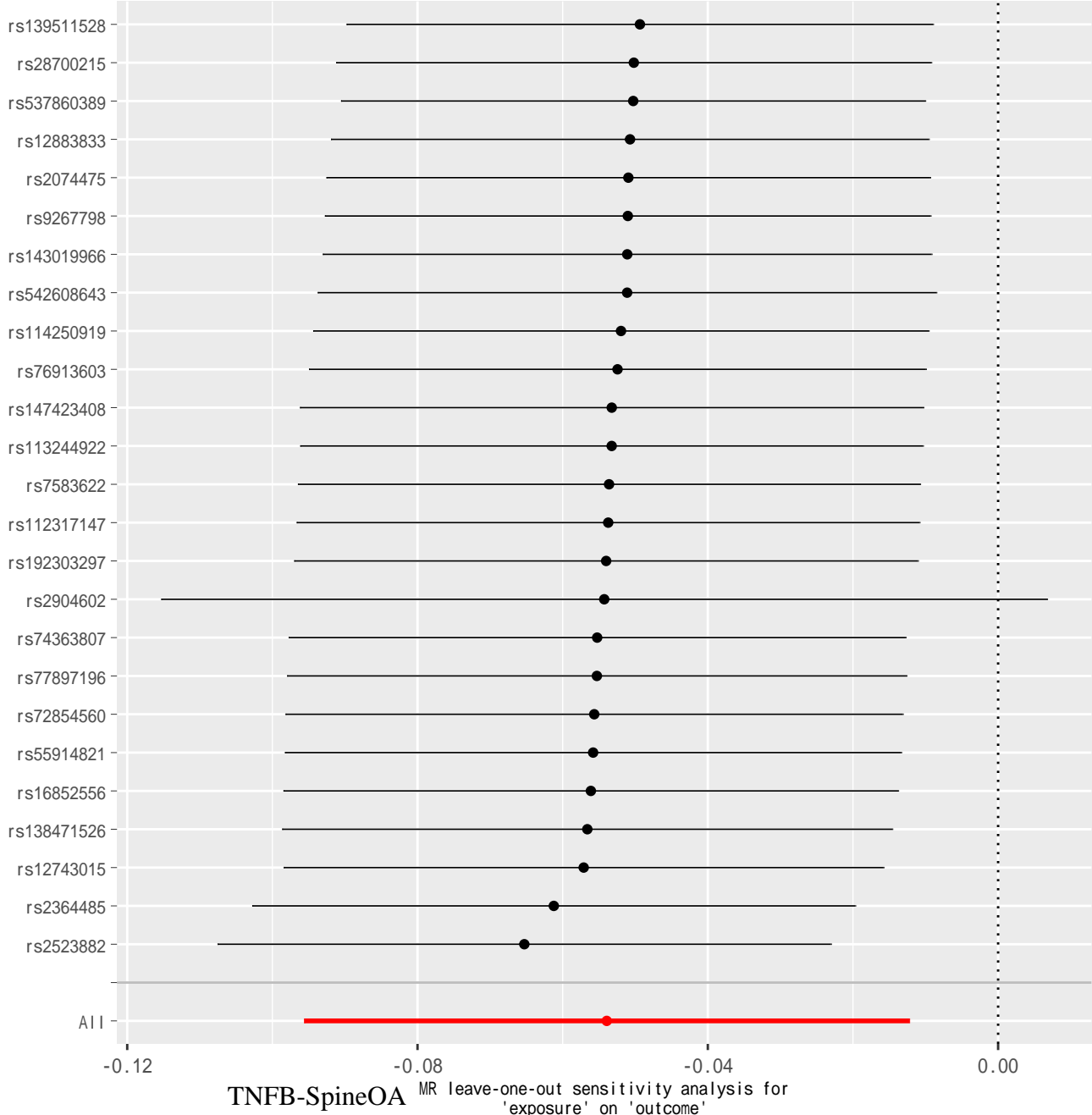

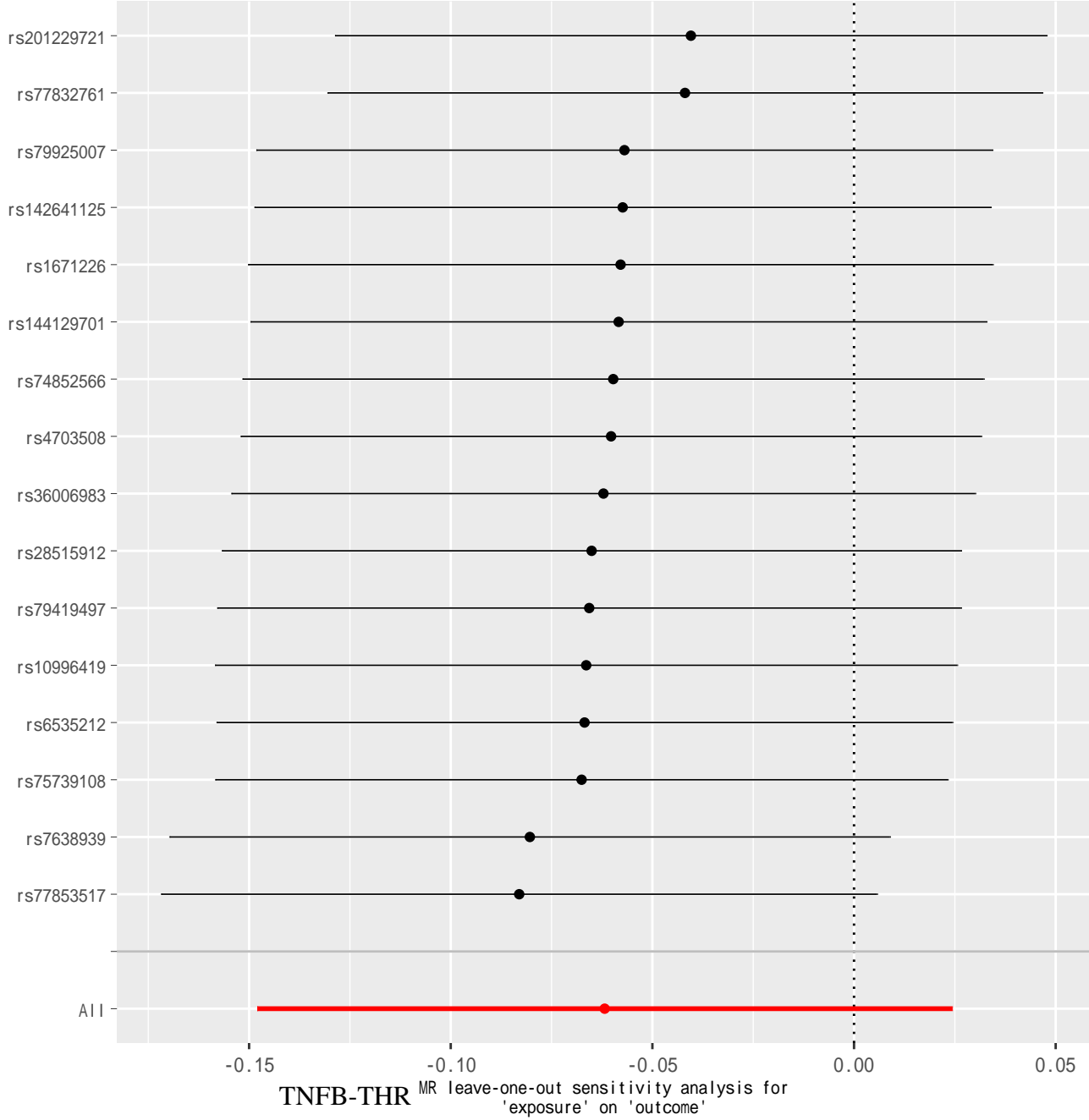

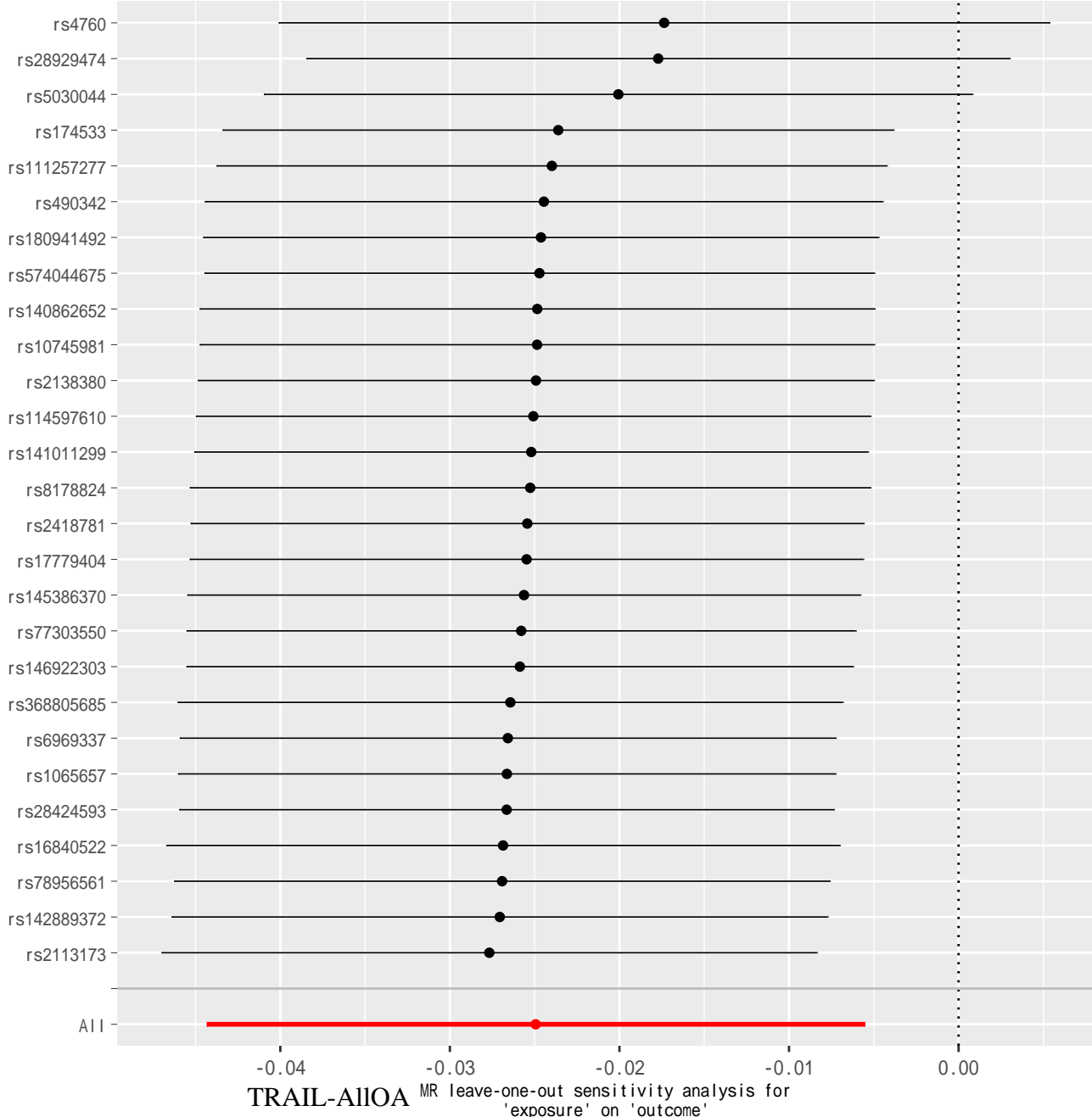

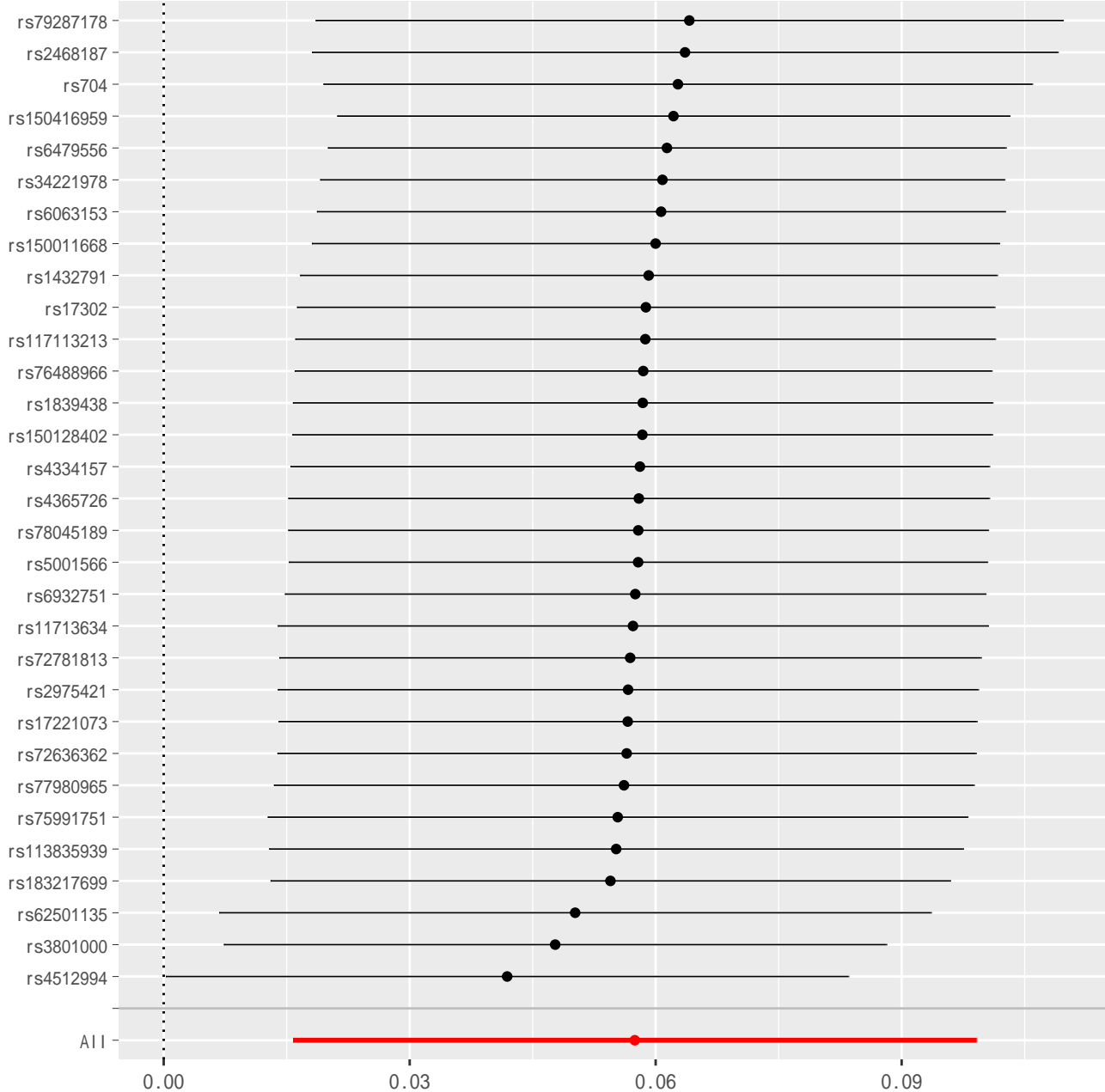

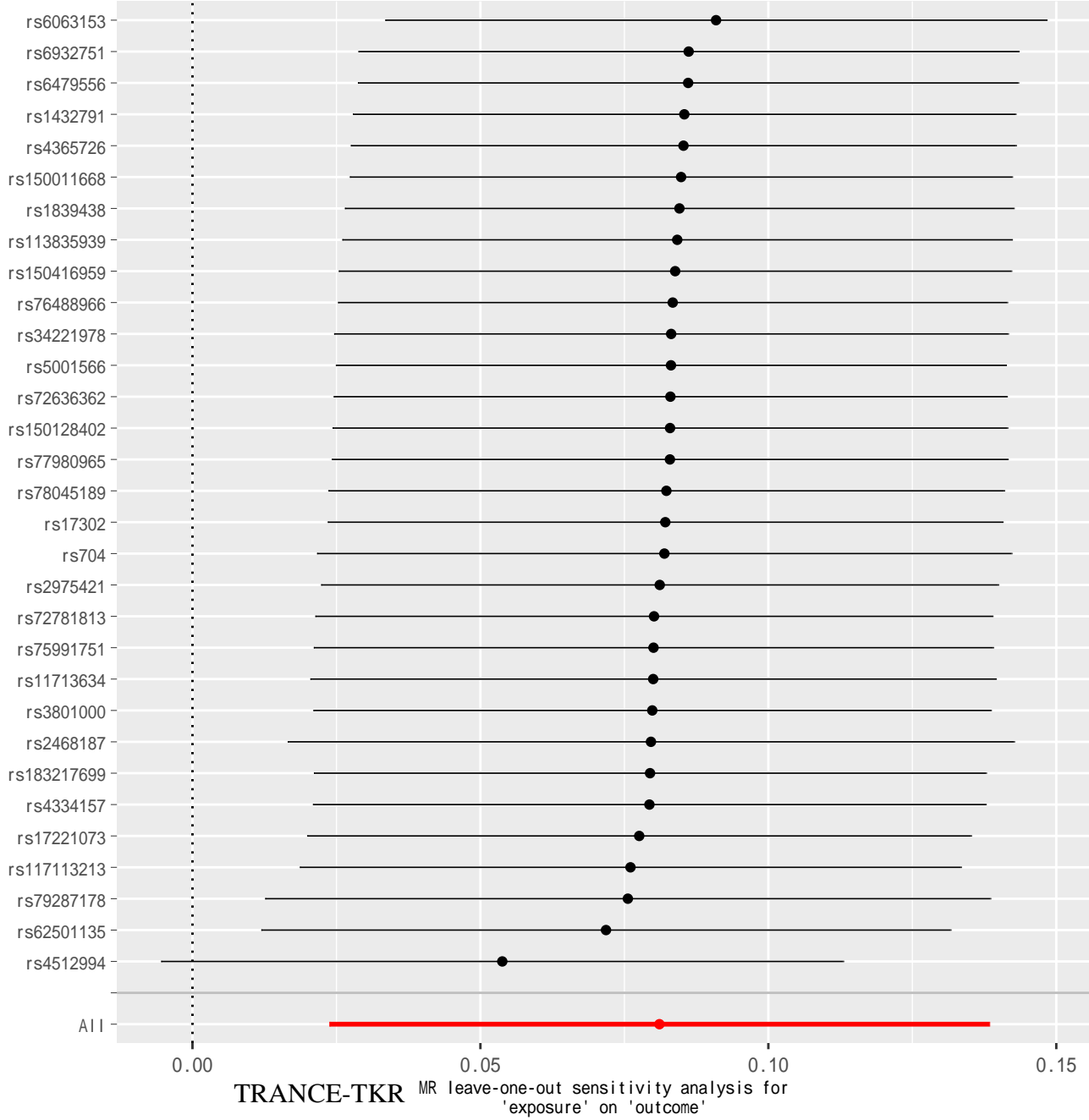

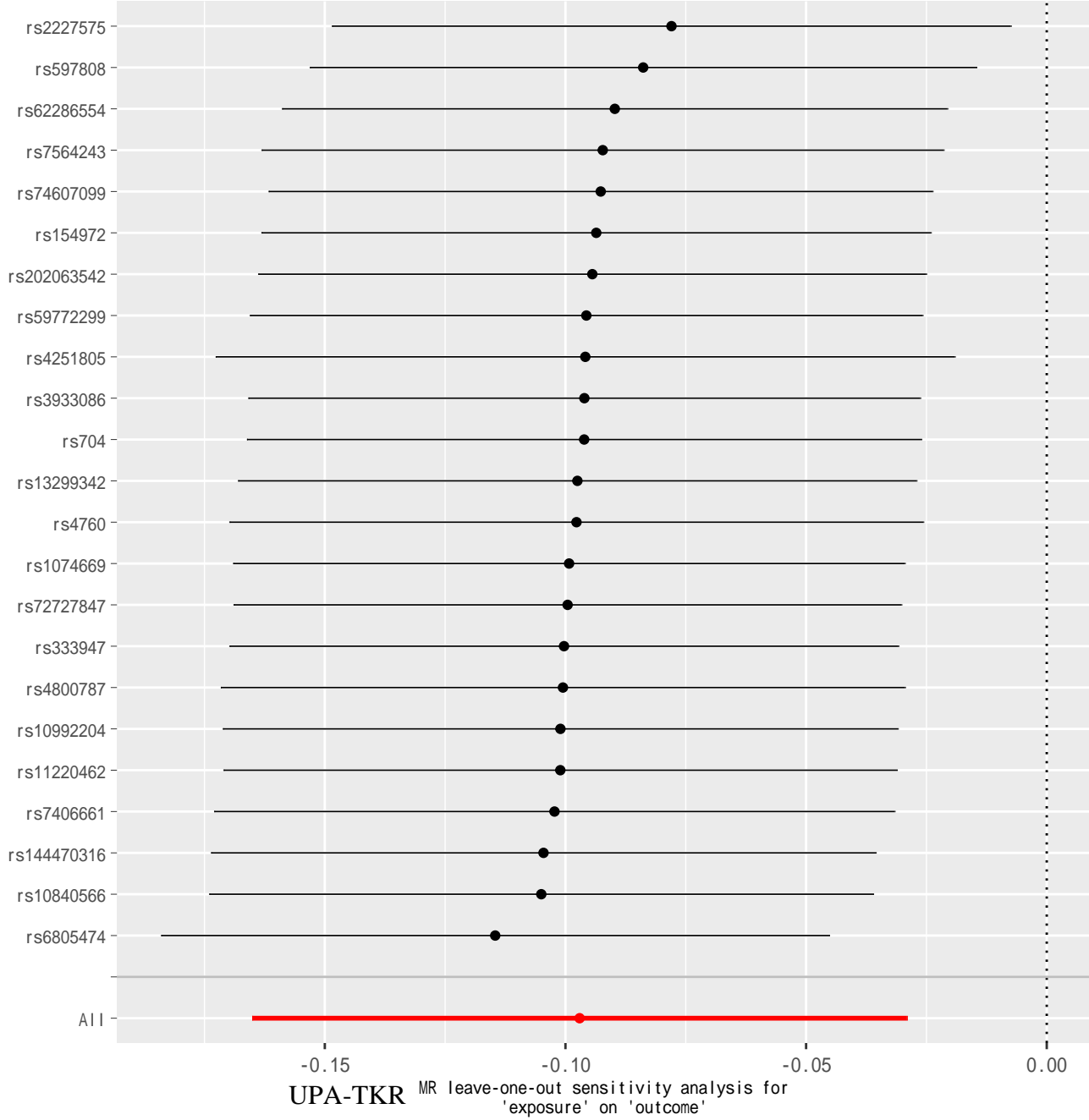

Supplement: Supplementary Figure 1 — Discovery of Leave One Out. [file DataSheet_1.pdf]
