# Supplementary material for: The causal effect of cytokine cycling levels on osteoarthritis: a bidirectional Mendelian randomized study
Source: Front Immunol. 2024 Jan 11;14:1334361. doi: 10.3389/fimmu.2023.1334361 (PMC10808687; doi:10.3389/fimmu.2023.1334361)
Supplement: Supplementary Figure 2 — Replication of Leave One Out. [file DataSheet_2.pdf]

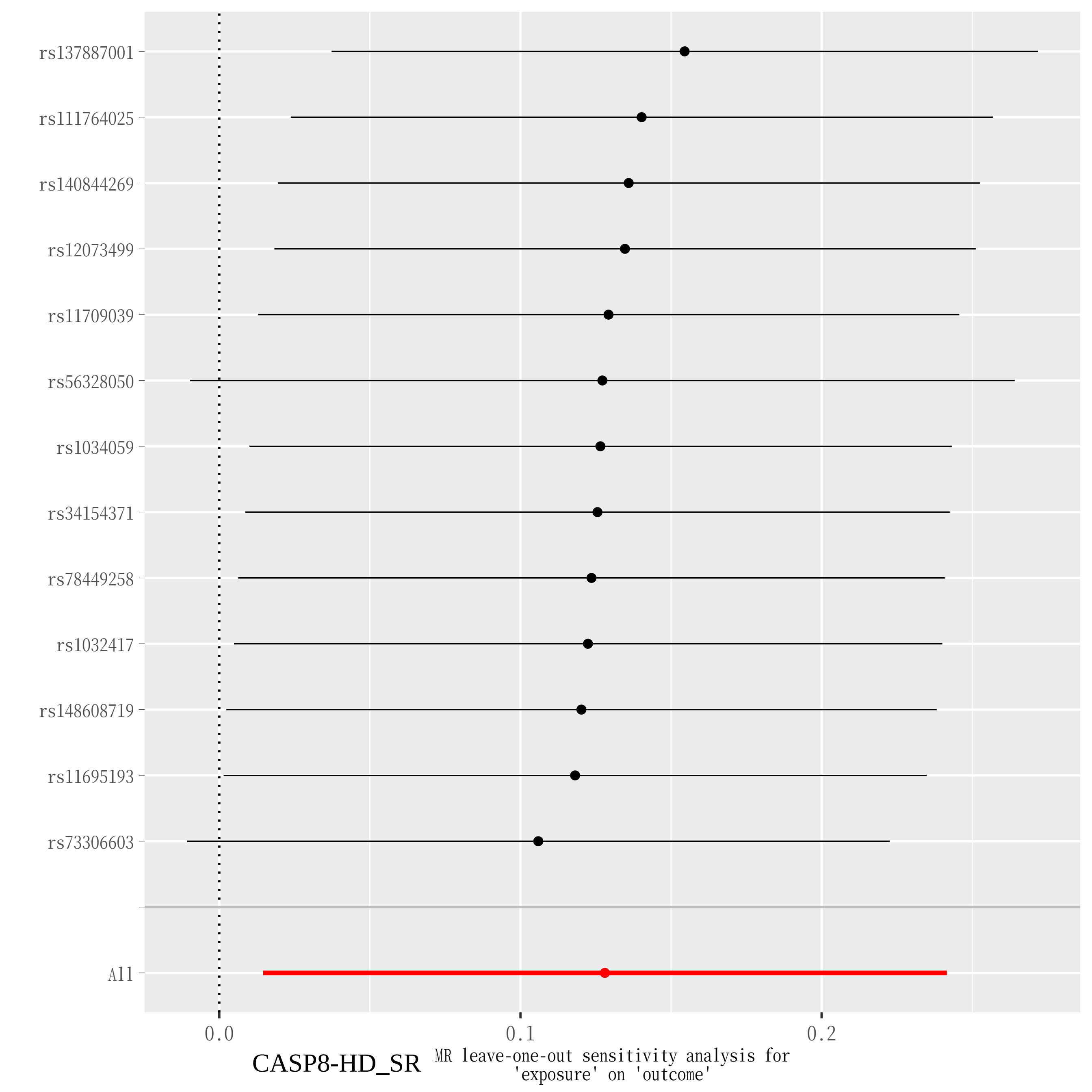
rs137887001
rs111764025
rs140844269
rs12073499
rs11709039
rs56328050
rs1034059
rs34154371
rs78449258
rs1032417
rs148608719
rs11695193
rs73306603
All
0.0
0.1
0.2
CASP8-HD_SR
MR leave-one-out sensitivity analysis for
'exposure' on 'outcome'

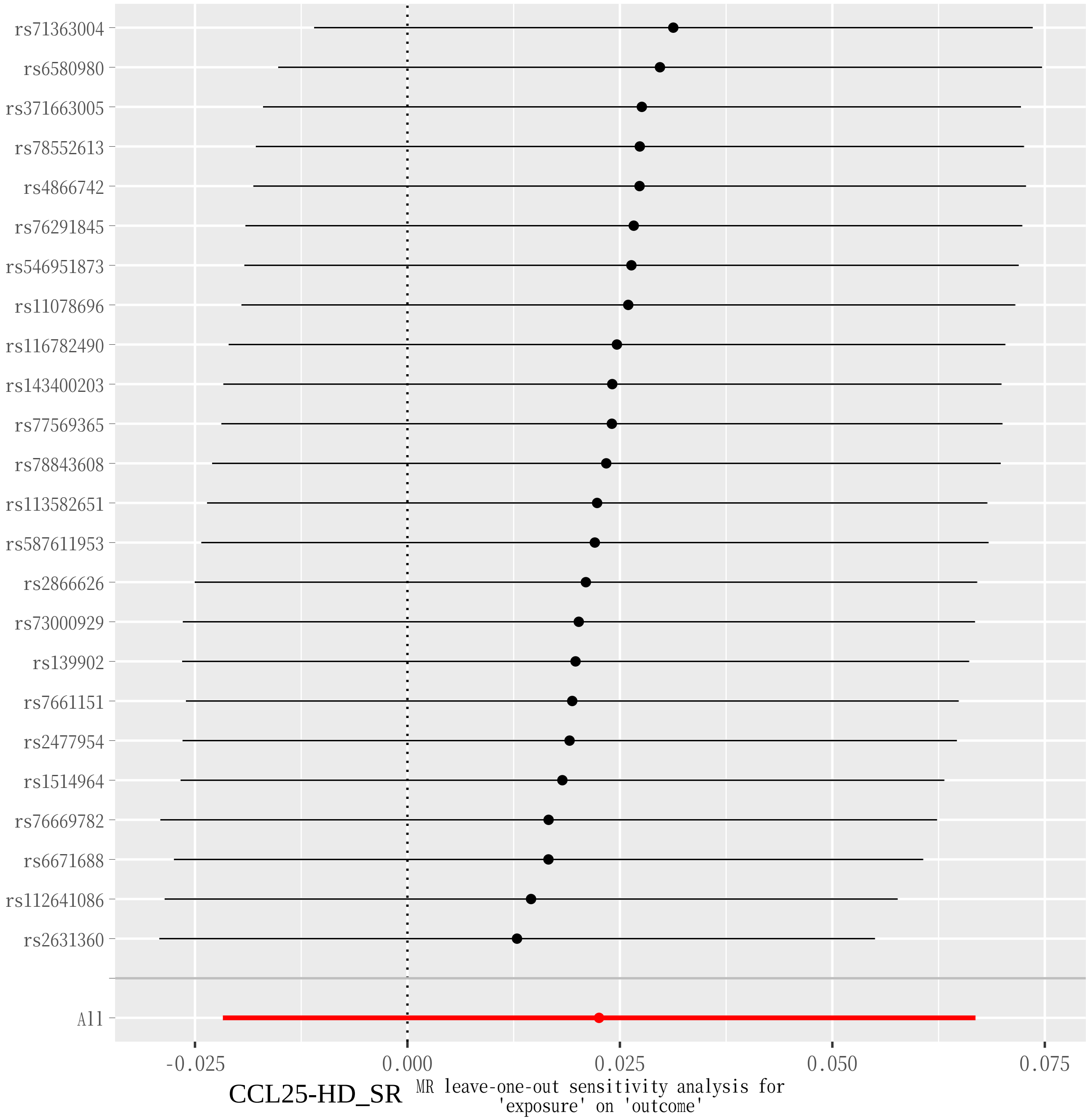

rs71363004
rs6580980
rs371663005
rs78552613
rs4866742
rs76291845
rs546951873
rs11078696
rs116782490
rs143400203
rs77569365
rs78843608
rs113582651
rs587611953
rs2866626
rs73000929
rs139902
rs7661151
rs2477954
rs1514964
rs76669782
rs6671688
rs112641086
rs2631360
All
-0.025
0.000
0.025
0.050
0.075
CCL25-HD_SR
MR leave-one-out sensitivity analysis for
'exposure' on 'outcome'

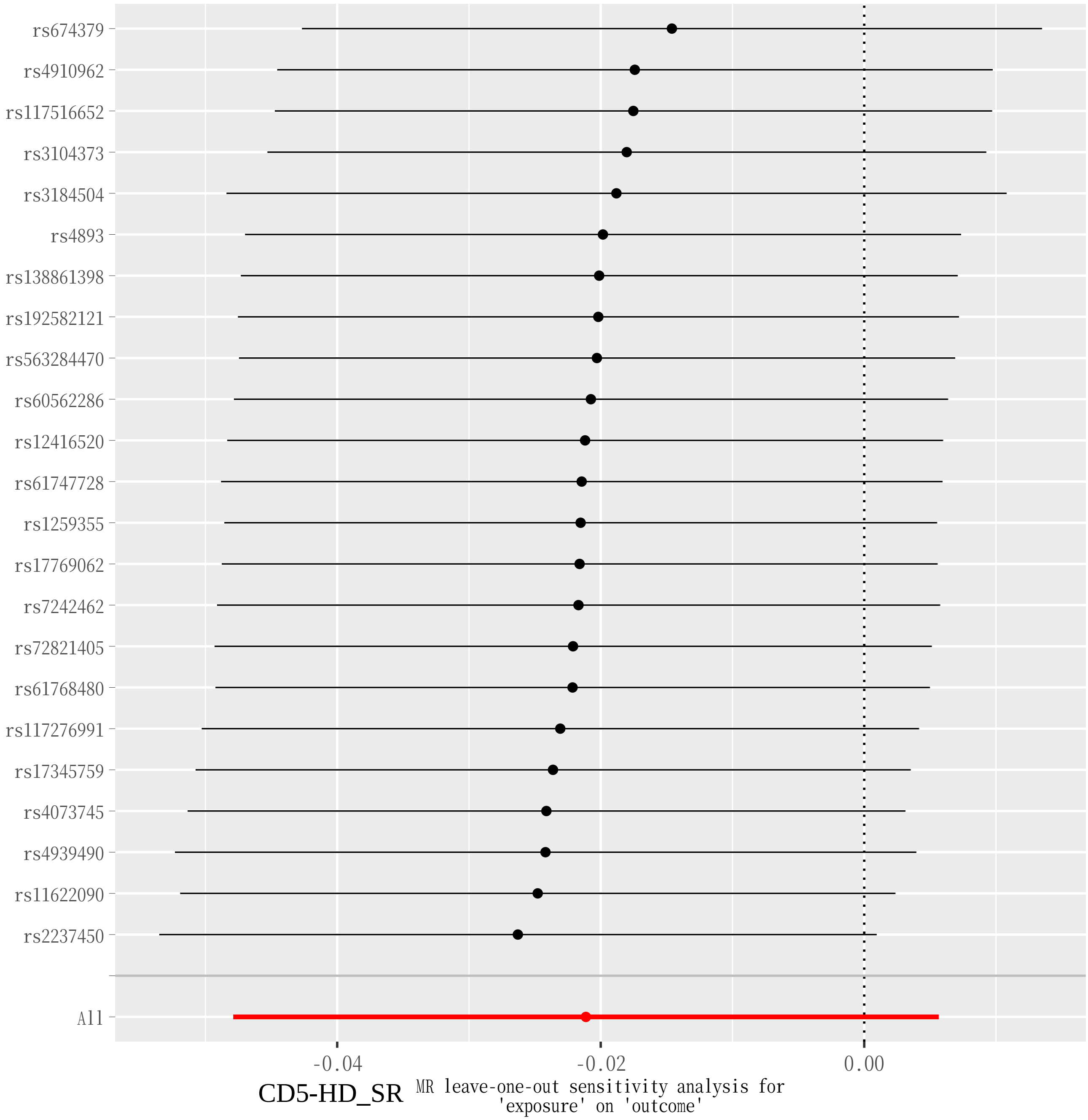

rs674379
rs4910962
rs117516652
rs3104373
rs3184504
rs4893
rs138861398
rs192582121
rs563284470
rs60562286
rs12416520
rs61747728
rs1259355
rs17769062
rs7242462
rs72821405
rs61768480
rs117276991
rs17345759
rs4073745
rs4939490
rs11622090
rs2237450
All
-0.04
-0.02
0.00
CD5-HD_SR
MR leave-one-out sensitivity analysis for
'exposure' on 'outcome'

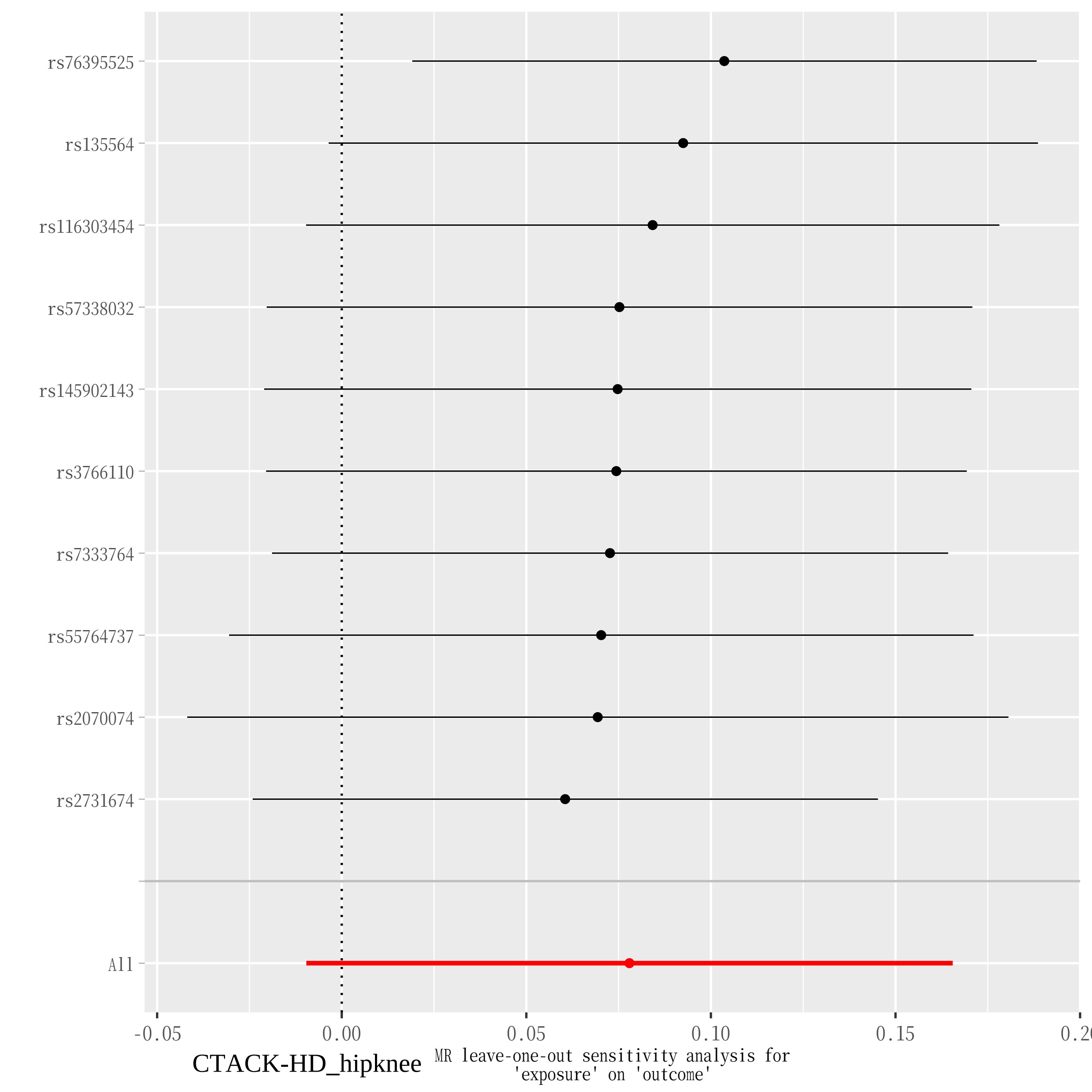
rs76395525
rs135564
rs116303454
rs57338032
rs145902143
rs3766110
rs7333764
rs55764737
rs2070074
rs2731674
All
-0.05
0.00
0.05
0.10
0.15
CTACK-HD_hipknee
MR leave-one-out sensitivity analysis for
'exposure' on 'outcome'

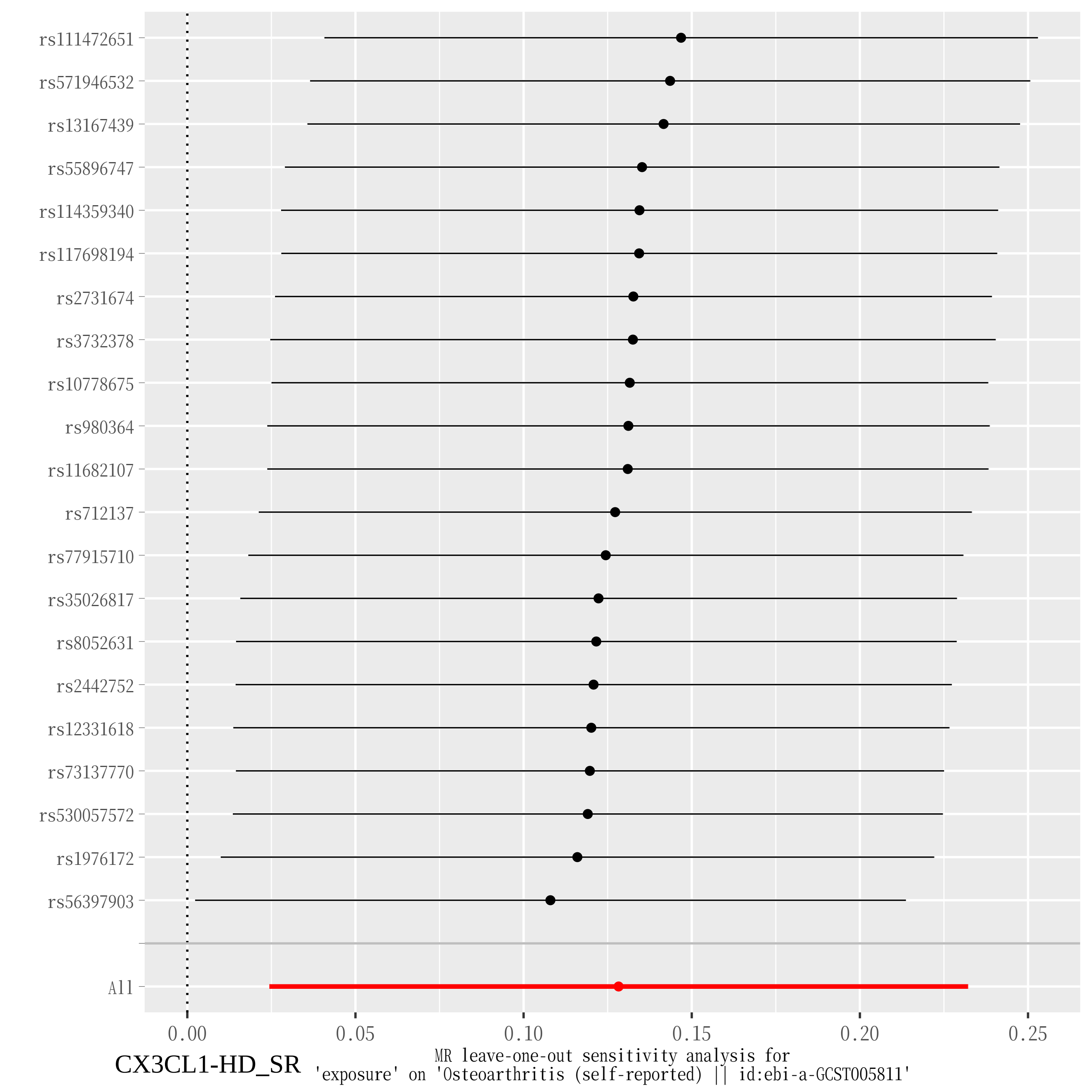
rs111472651
rs571946532
rs13167439
rs55896747
rs114359340
rs117698194
rs2731674
rs3732378
rs10778675
rs980364
rs11682107
rs712137
rs77915710
rs35026817
rs8052631
rs2442752
rs12331618
rs73137770
rs530057572
rs1976172
rs56397903
All
0.00
0.05
0.10
0.15
0.20
0.25
CX3CL1-HD_SR
MR leave-one-out sensitivity analysis for
'exposure' on 'Osteoarthritis (self-reported) || id:ebi-a-GCST005811'

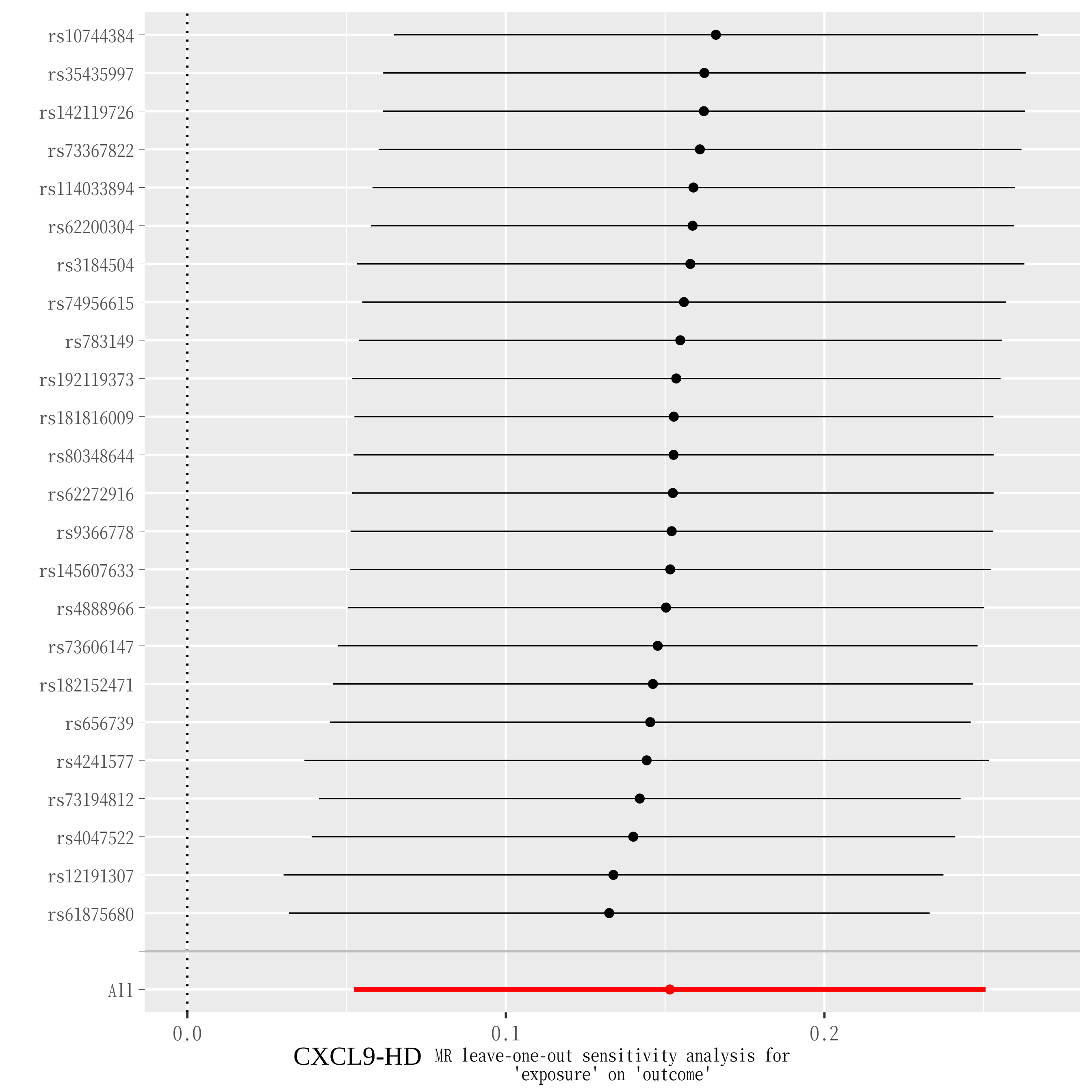

rs10744384
rs35435997
rs142119726
rs73367822
rs114033894
rs62200304
rs3184504
rs74956615
rs783149
rs192119373
rs181816009
rs80348644
rs62272916
rs9366778
rs145607633
rs4888966
rs73606147
rs182152471
rs656739
rs4241577
rs73194812
rs4047522
rs12191307
rs61875680
All
0.0
0.1
0.2
CXCL9-HD
MR leave-one-out sensitivity analysis for
'exposure' on 'outcome'

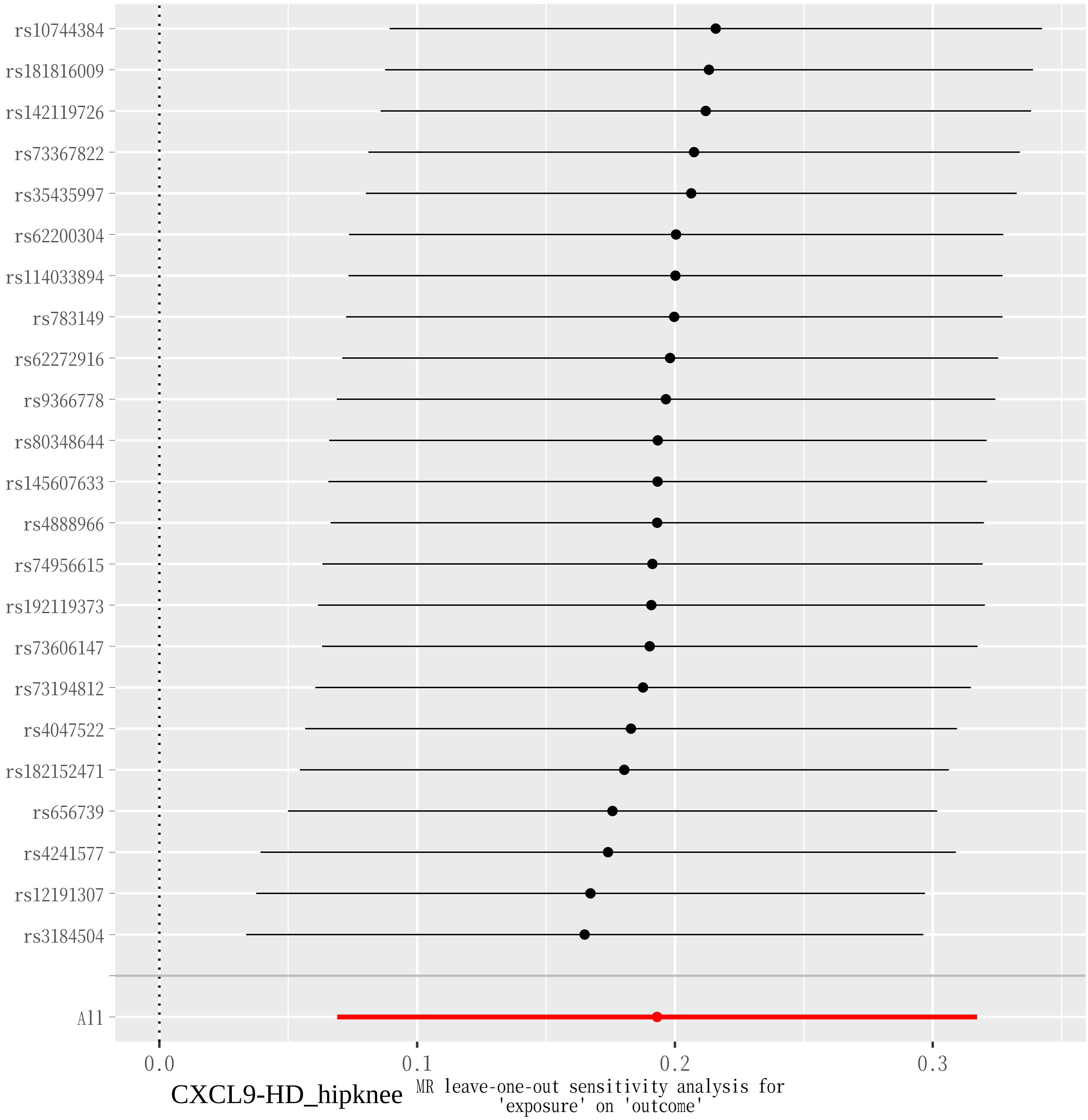

CXCL9-HD_hipknee

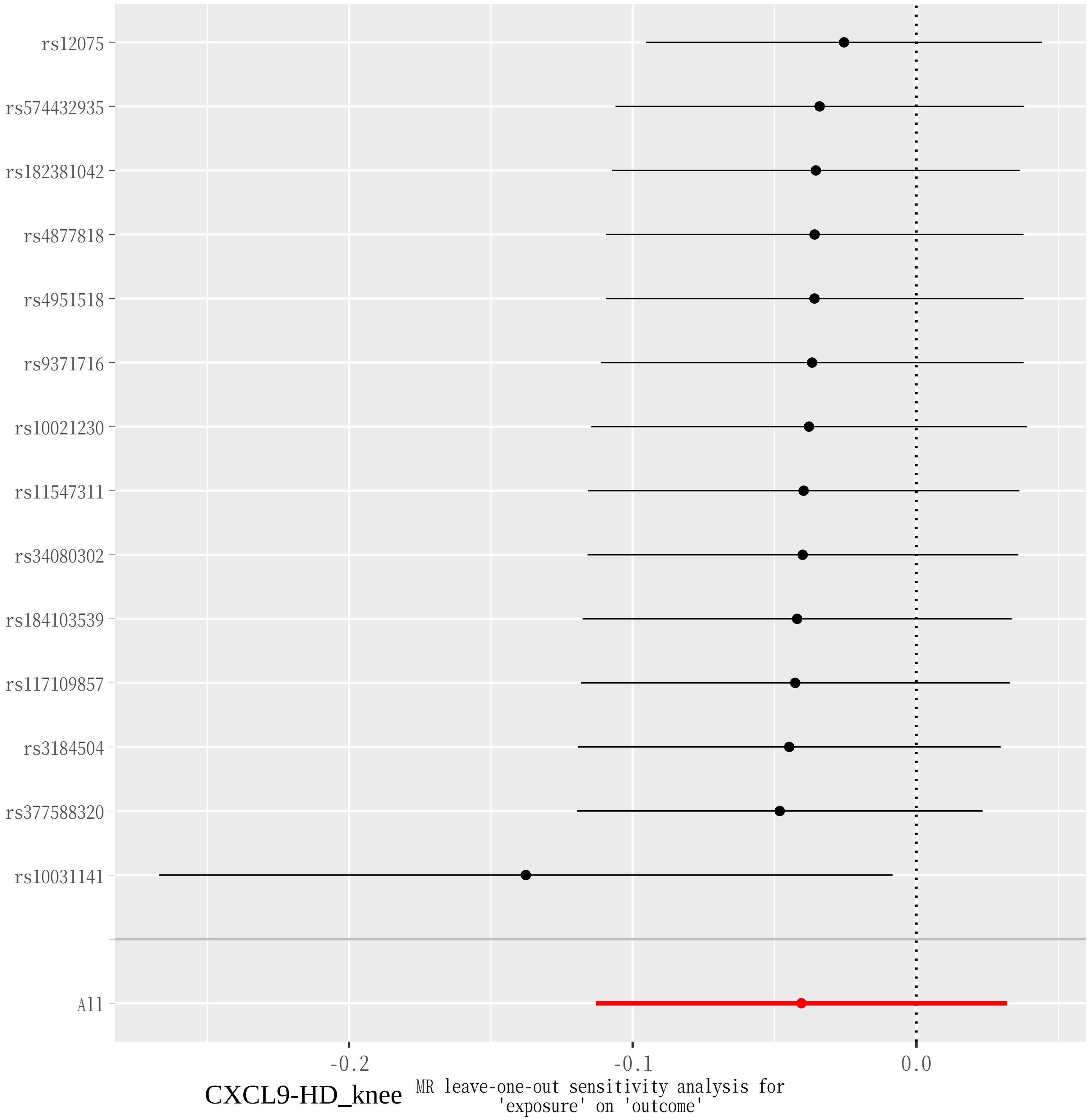

CXCL9-HD_knee

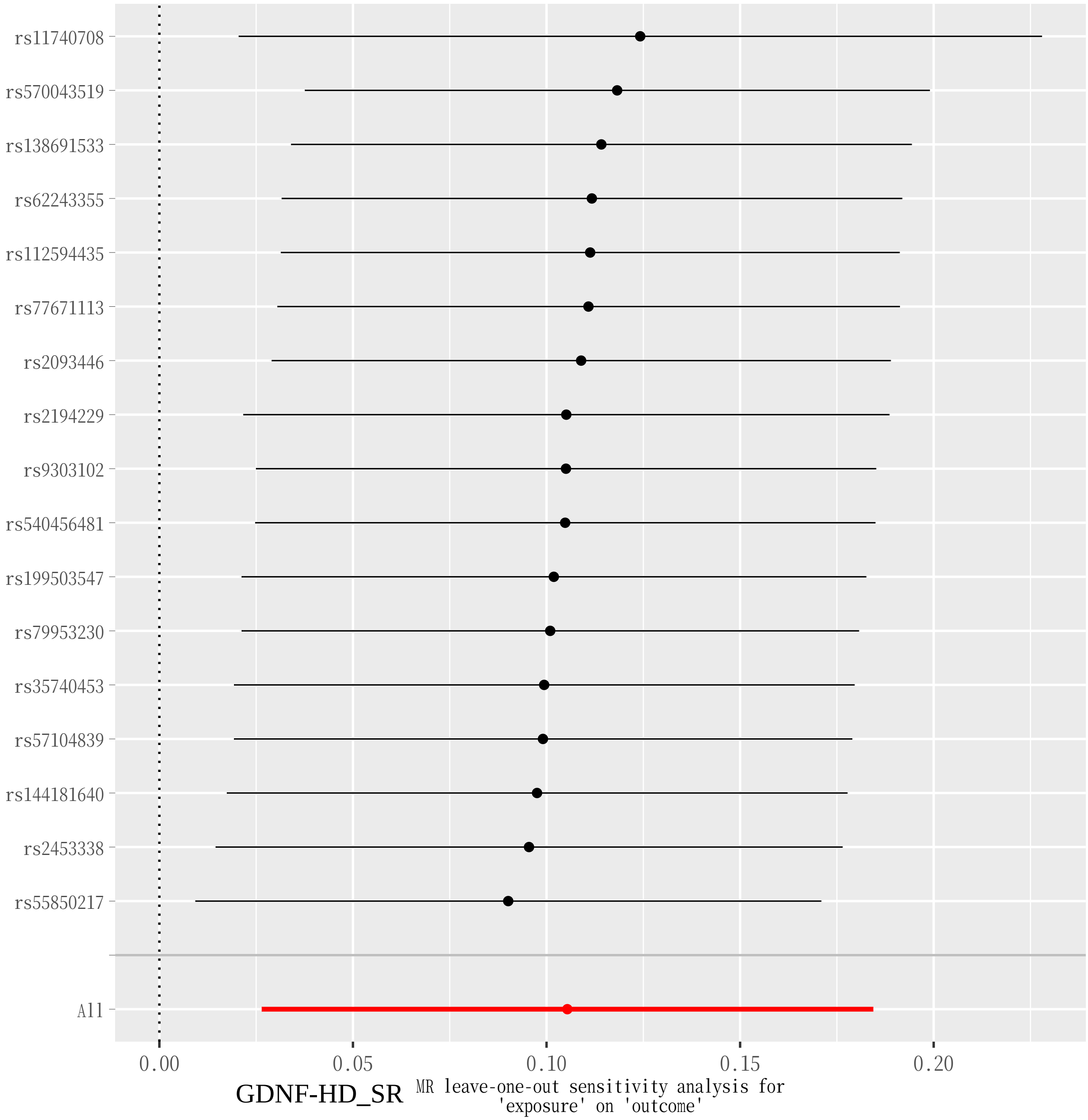

rs11740708
rs570043519
rs138691533
rs62243355
rs112594435
rs77671113
rs2093446
rs2194229
rs9303102
rs540456481
rs199503547
rs79953230
rs35740453
rs57104839
rs144181640
rs2453338
rs55850217
All
0.00
0.05
0.10
0.15
0.20
GDNF-HD_SR
MR leave-one-out sensitivity analysis for
'exposure' on 'outcome'

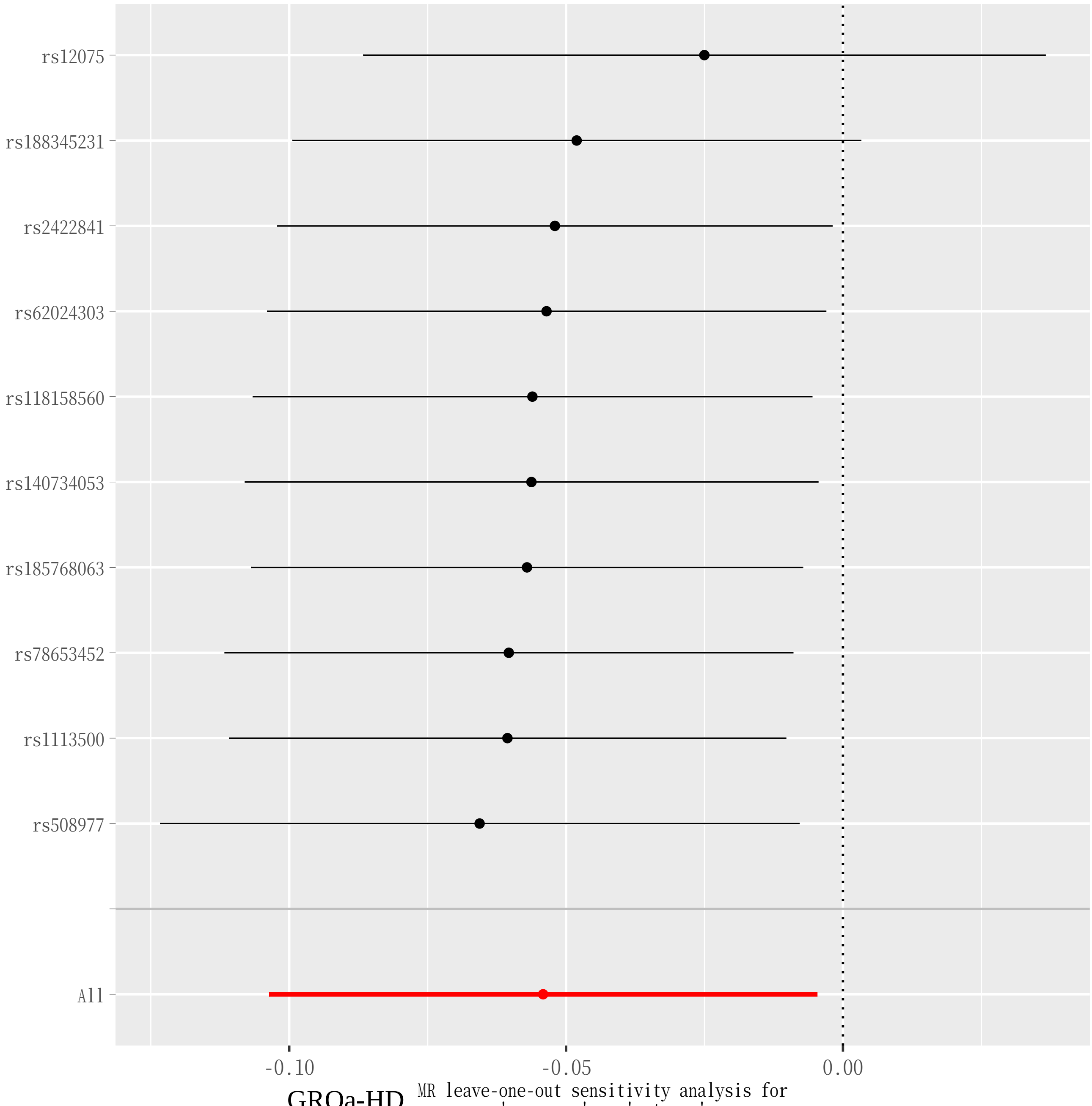
rs12075
rs188345231
rs2422841
rs62024303
rs118158560
rs140734053
rs185768063
rs78653452
rs1113500
rs508977
All
-0.10
-0.05
0.00
MR leave-one-out sensitivity analysis for
'exposure' on 'outcome'

GROa-HD

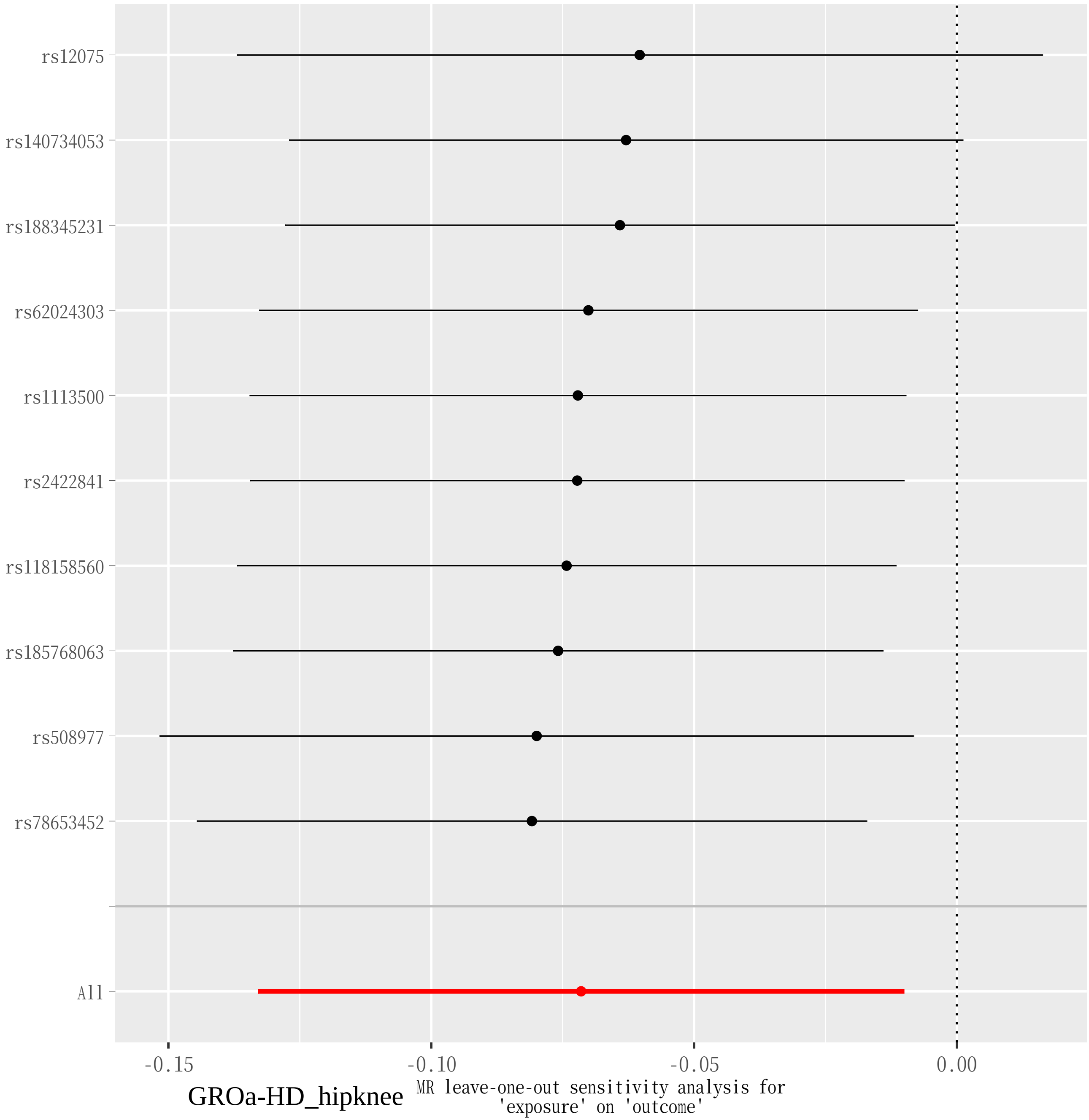

GROa-HD_hipknee

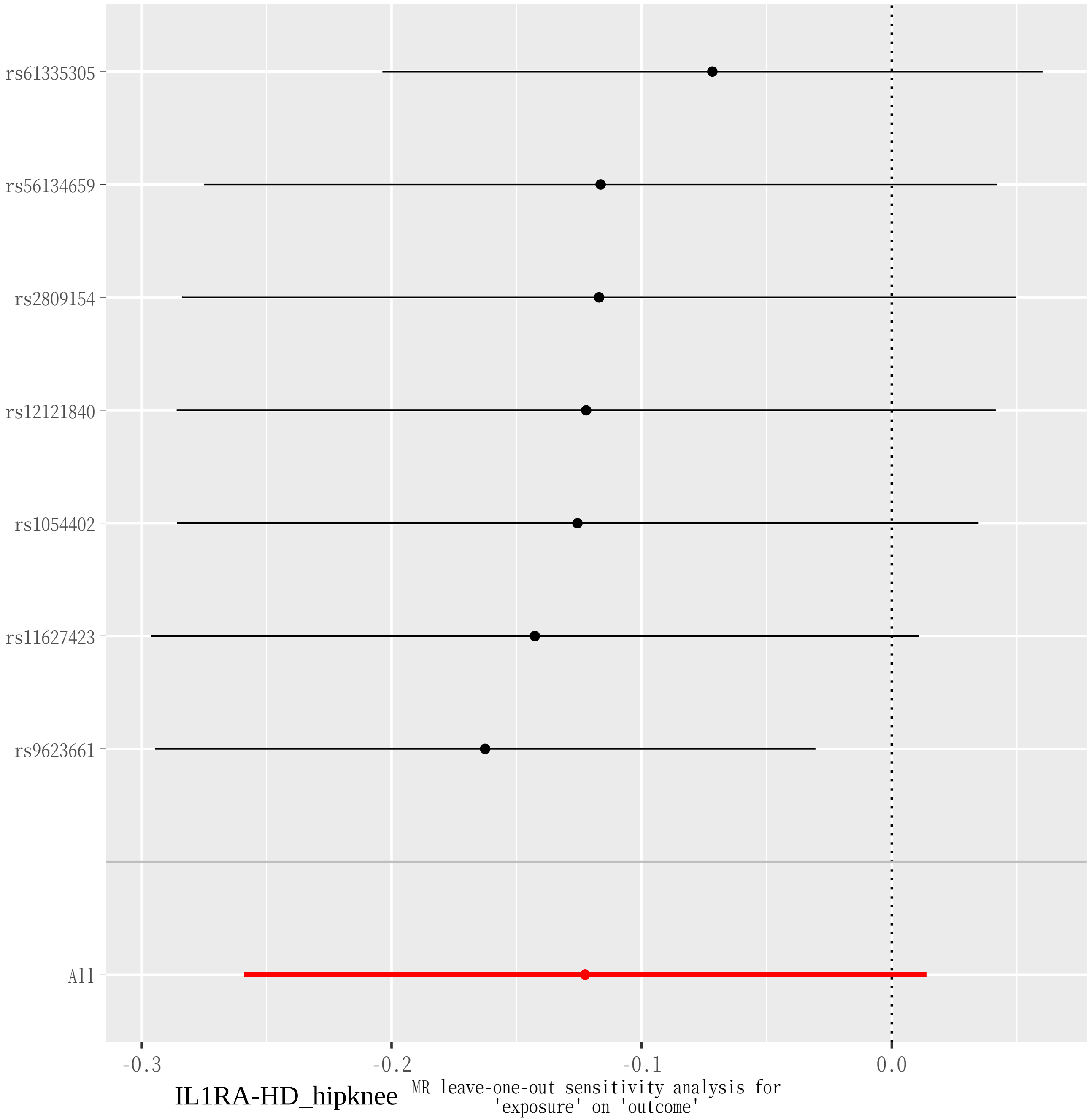

IL1RA-HD_hipknee

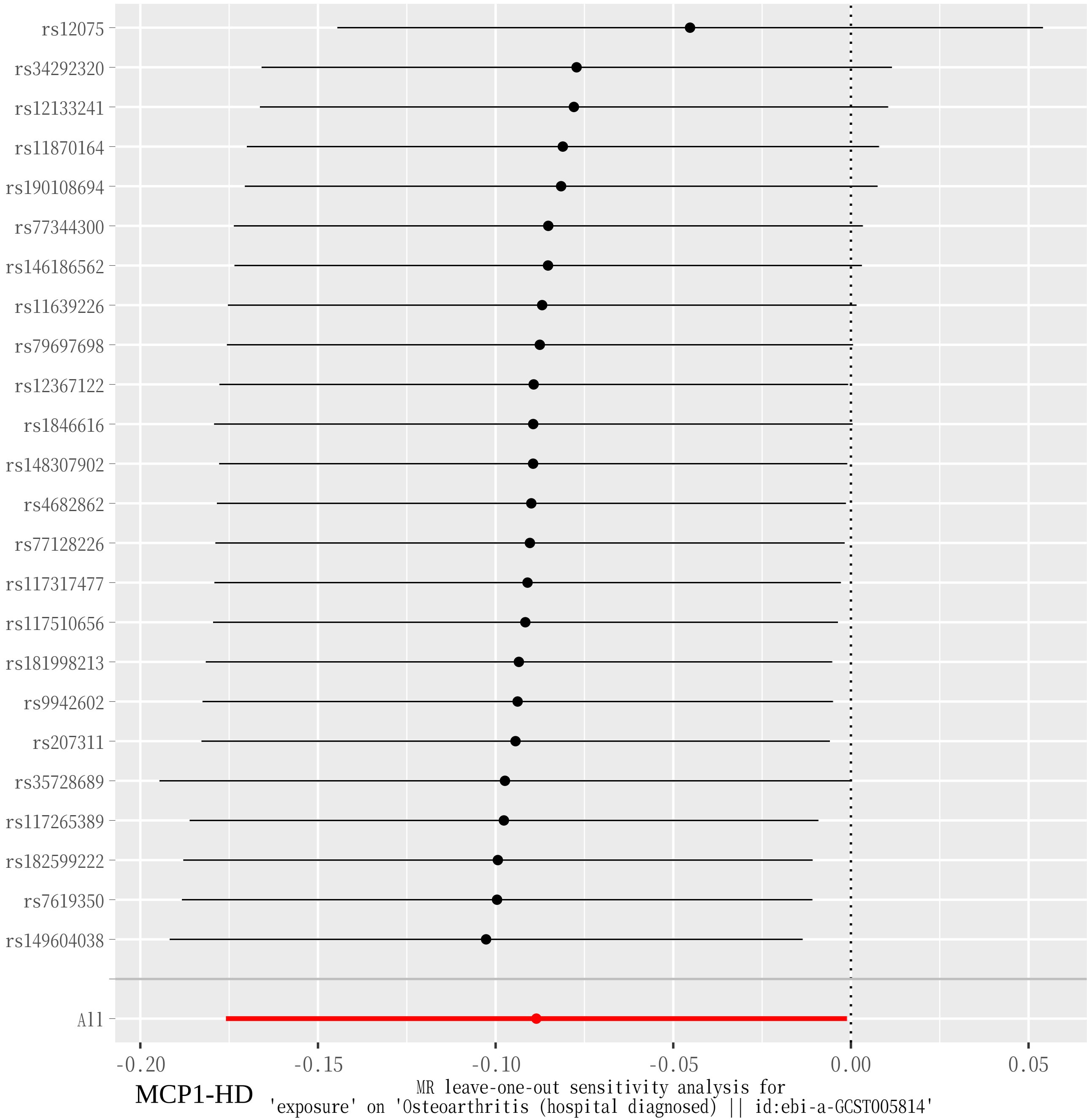
rs12075
rs34292320
rs12133241
rs11870164
rs190108694
rs77344300
rs146186562
rs11639226
rs79697698
rs12367122
rs1846616
rs148307902
rs4682862
rs77128226
rs117317477
rs117510656
rs181998213
rs9942602
rs207311
rs35728689
rs117265389
rs182599222
rs7619350
rs149604038
All
-0.20
-0.15
-0.10
-0.05
0.00
0.05
MR leave-one-out sensitivity analysis for
'exposure' on 'Osteoarthritis (hospital diagnosed) || id:ebi-a-GCST005814'
MCP1-HD

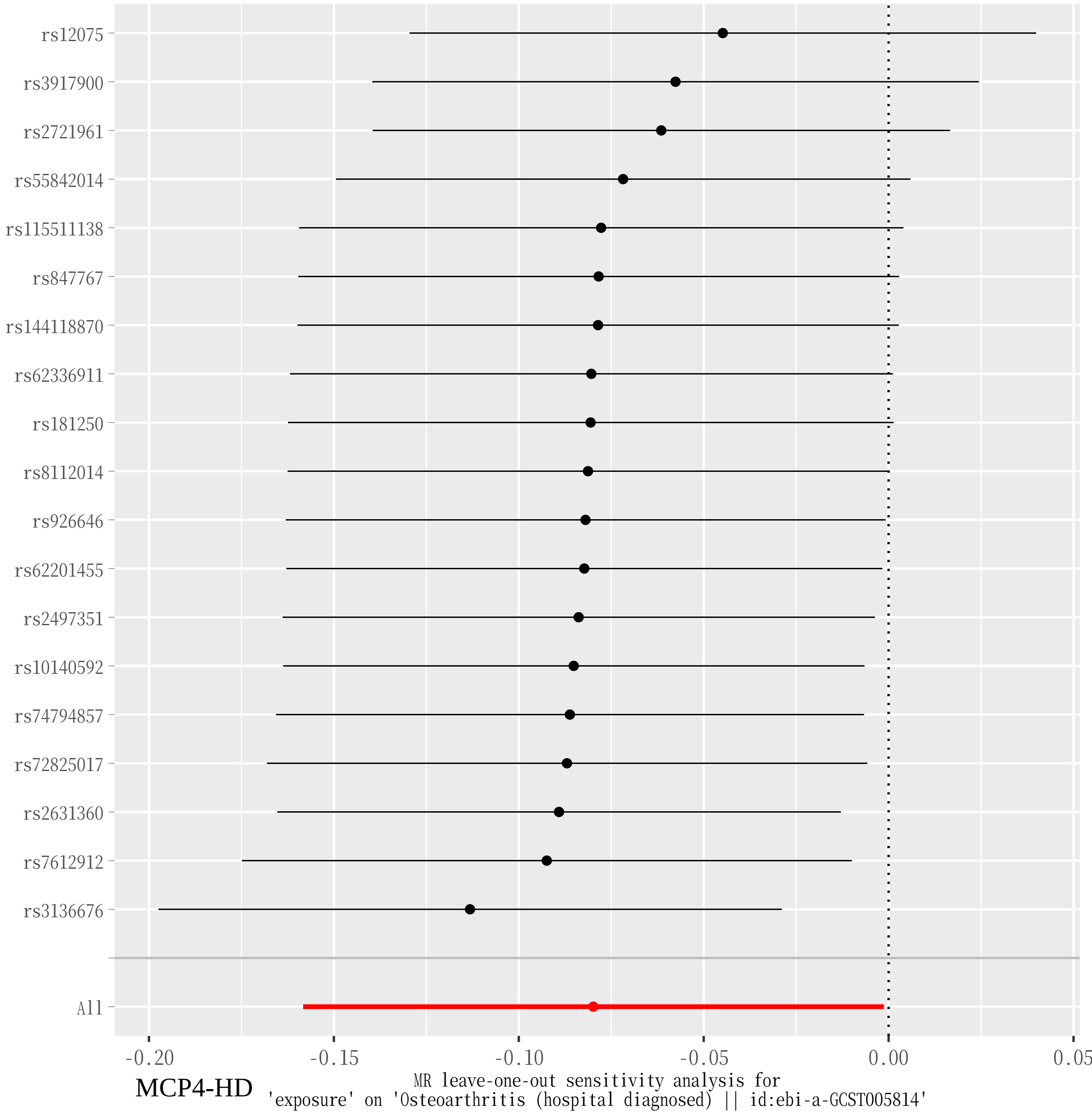
rs12075
rs3917900
rs2721961
rs55842014
rs115511138
rs847767
rs144118870
rs62336911
rs181250
rs8112014
rs926646
rs62201455
rs2497351
rs10140592
rs74794857
rs72825017
rs2631360
rs7612912
rs3136676
All
-0.20
-0.15
-0.10
-0.05
0.00
0.05
MR leave-one-out sensitivity analysis for
'exposure' on 'Osteoarthritis (hospital diagnosed) || id:ebi-a-GCST005814'

MCP4-HD

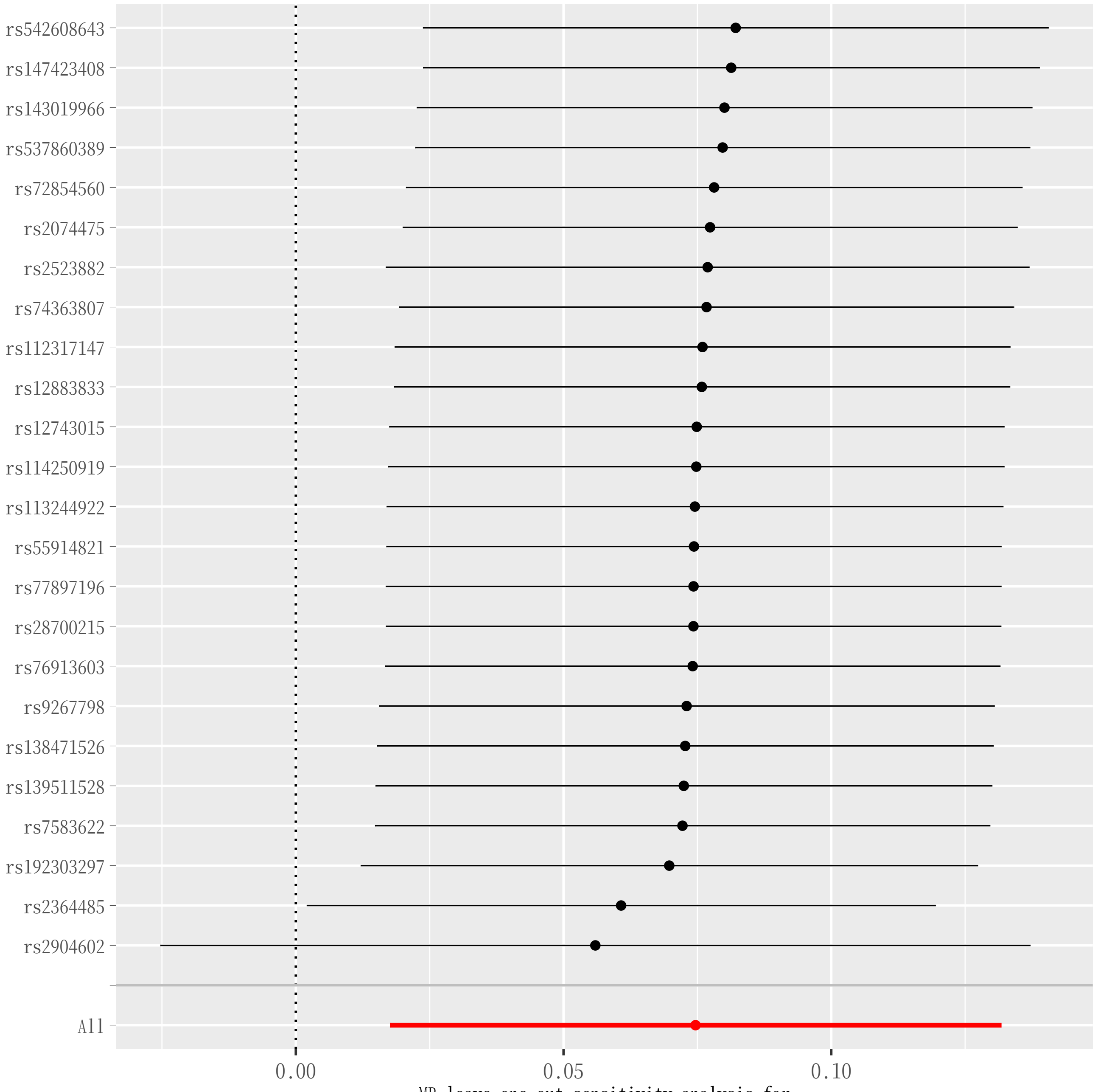

TNFB-HD

MR leave-one-out sensitivity analysis for
'exposure' on 'Osteoarthritis (hospital diagnosed) || id:ebi-a-GCST005814'

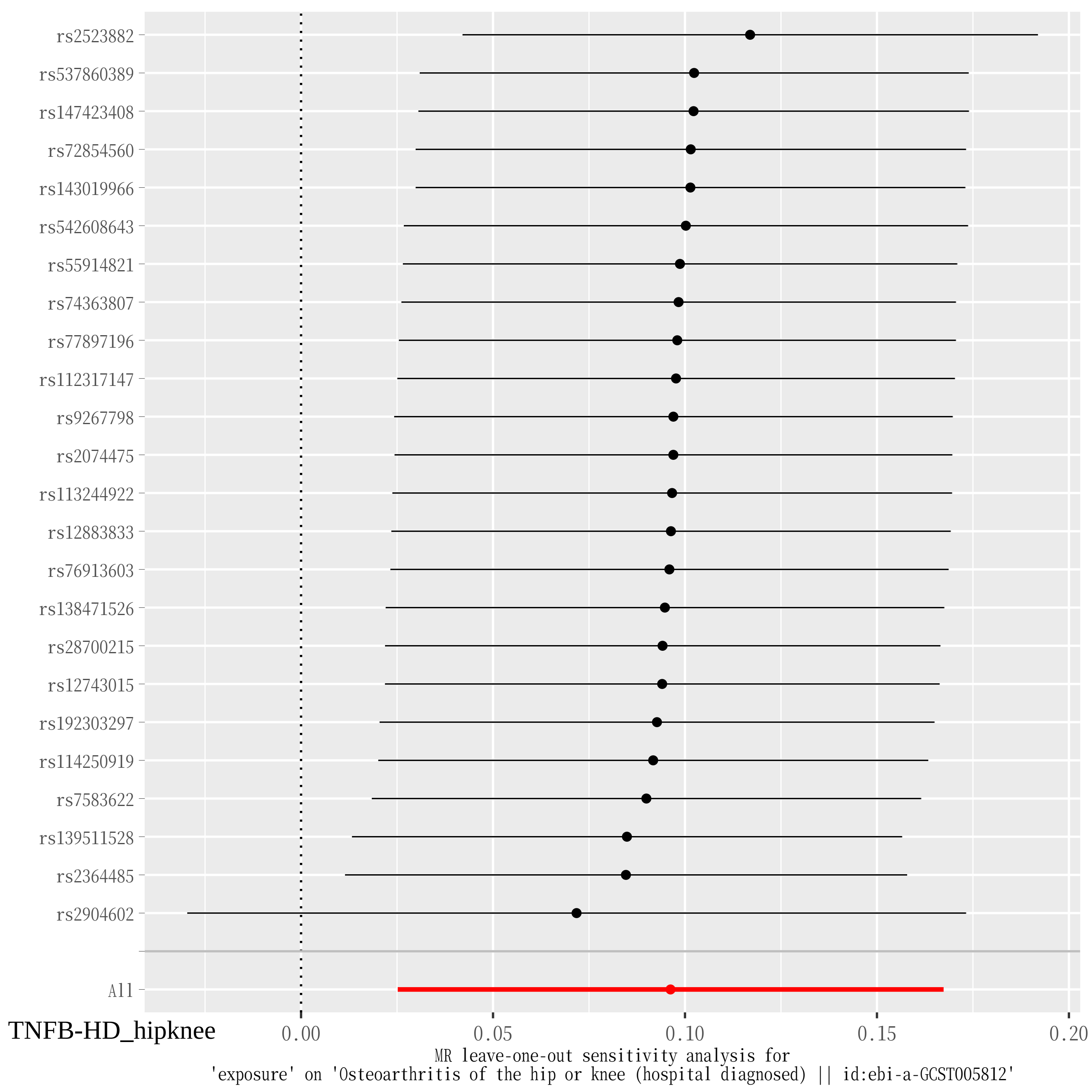
rs2523882
rs537860389
rs147423408
rs72854560
rs143019966
rs542608643
rs55914821
rs74363807
rs77897196
rs112317147
rs9267798
rs2074475
rs113244922
rs12883833
rs76913603
rs138471526
rs28700215
rs12743015
rs192303297
rs114250919
rs7583622
rs139511528
rs2364485
rs2904602
All
TNFB-HD_hipknee
0.00
0.05
0.10
0.15
0.20
MR leave-one-out sensitivity analysis for
'exposure' on 'Osteoarthritis of the hip or knee (hospital diagnosed) || id:ebi-a-GCST005812'

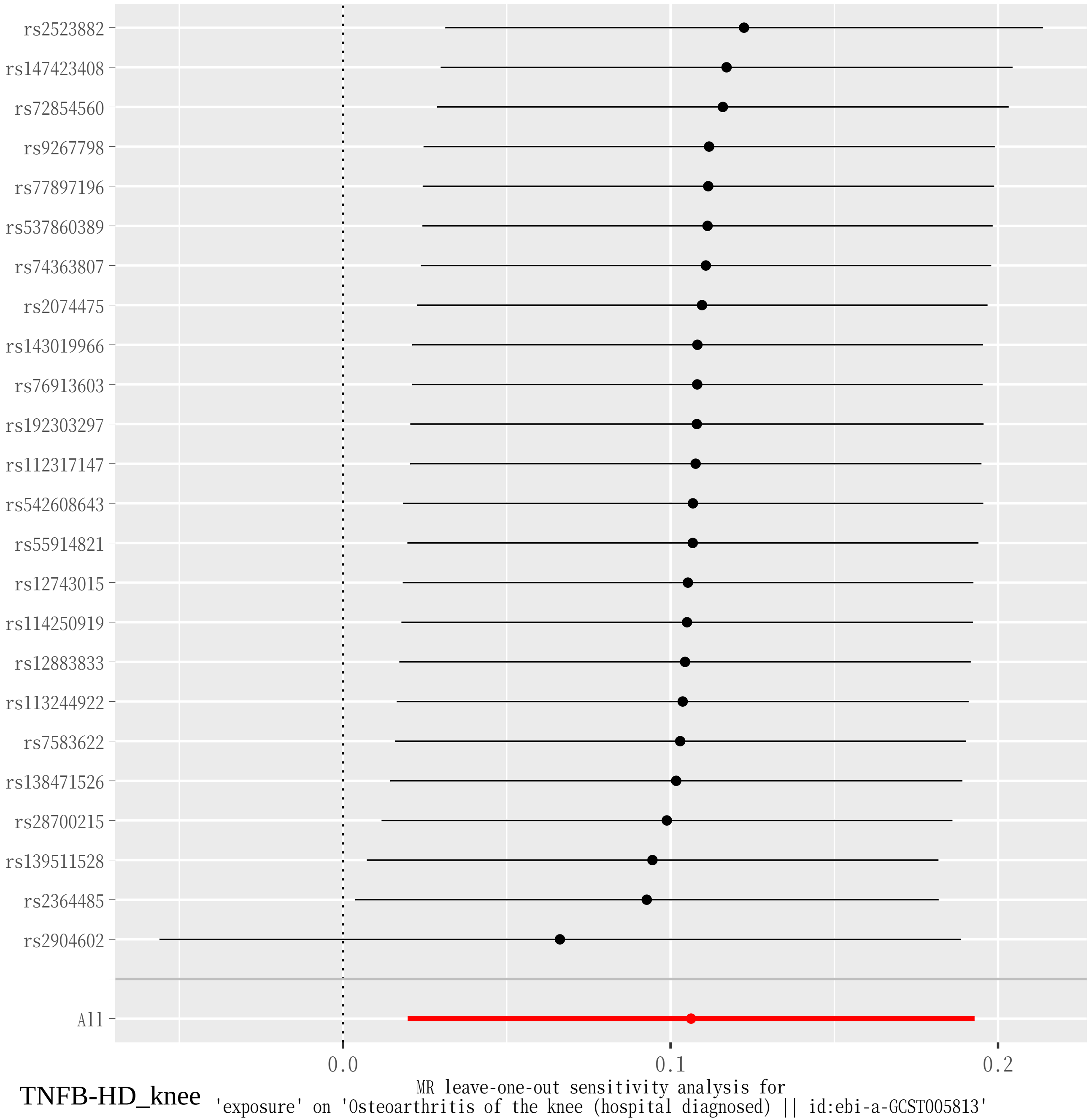
rs2523882
rs147423408
rs72854560
rs9267798
rs77897196
rs537860389
rs74363807
rs2074475
rs143019966
rs76913603
rs192303297
rs112317147
rs542608643
rs55914821
rs12743015
rs114250919
rs12883833
rs113244922
rs7583622
rs138471526
rs28700215
rs139511528
rs2364485
rs2904602
All
0.0
0.1
0.2
MR leave-one-out sensitivity analysis for
'exposure' on 'Osteoarthritis of the knee (hospital diagnosed) || id:ebi-a-GCST005813'

TNFB-HD_knee
